# EGF-Upregulated lncRNA ESSENCE Promotes Colorectal Cancer Growth through Stabilizing CAD and Ferroptosis Defense

**DOI:** 10.34133/research.0649

**Published:** 2025-04-03

**Authors:** Xiaoshan Xie, Boyu Zhang, Jingxuan Peng, Ning Ma, Qihao Pan, Yue Wei, Huilin Jin, Fenghai Yu, Xiaoling Huang, Peng Zhang, Jiarui Wang, Jiaying Zheng, Xiaofang Ying, Ran-yi Liu, Hongyan Yu, Mong-Hong Lee, Xiangqi Meng

**Affiliations:** ^1^ Department of General Surgery, The Sixth Affiliated Hospital, Sun Yat-sen University, Guangzhou 510655, China.; ^2^ Guangdong Provincial Key Laboratory of Colorectal and Pelvic Floor Diseases, Guangdong Institute of Gastroenterology, The Sixth Affiliated Hospital, Sun Yat-sen University, Guangzhou 510655, China.; ^3^ Biomedical Innovation Center, The Sixth Affiliated Hospital, Sun Yat-sen University, Guangzhou 510655, China.; ^4^ Department of Obstetrics and Gynecology, The Sixth Affiliated Hospital, Sun Yat-sen University, Guangzhou 510655, China.; ^5^ Department of Hepatobiliary, Pancreatic and Splenic Surgery, The Sixth Affiliated Hospital, Sun Yat-sen University, Guangzhou 510655, China.; ^6^ Department of Radiation Oncology, Hubei Cancer Hospital, Tongji Medical College, Huazhong University of Science and Technology, Wuhan 430079, China.; ^7^ State Key Laboratory of Oncology in South China & Collaborative Innovation Center for Cancer Medicine, Sun Yat-sen University Cancer Center, Guangzhou 510060, China.; ^8^ Department of Clinical Biological Resource Bank, Guangzhou Institute of Pediatrics, Guangzhou Women and Children’s Medical Center, Guangzhou Medical University, Guangzhou 510623, China.

## Abstract

Epidermal growth factor receptor/mitogen-activated protein kinase (EGFR/MAPK) signaling is highly activated in various types of cancer. The long noncoding RNAs induced by this pathway and their roles in colorectal cancer (CRC) have not been fully elucidated. In this study, based on the profiling of long noncoding RNAs triggered by EGFR/MAPK signaling, we identified that ESSENCE (EGF [epidermal growth factor] Signal Sensing CAD’s Effect; ENST00000415336), which is mediated by the transcription factor early growth response factor 1, functions as a potent oncogenic molecule that predicts poor prognosis in CRC. Mechanistically, ESSENCE directly interacts with carbamoyl-phosphate synthetase 2, aspartate transcarbamylase, and dihydroorotase (CAD) and competitively attenuates CAD degradation mediated by its newly discovered E3 ligase KEAP1, thereby suppressing ferroptosis and promoting CRC progression. Importantly, combinational treatment of the mitogen-activated extracellular signal-regulated kinase inhibitor selumetinib and ferroptosis inducer sulfasalazine synergistically suppresses ESSENCE-high CRC in a patient-derived xenograft mouse model. Taken together, these findings demonstrate the crucial role of ESSENCE in mediating CRC progression by regulating CAD stabilization and suggest a therapeutic strategy of targeting the ESSENCE–CAD axis in CRC.

## Introduction

Colorectal cancer (CRC) poses a marked challenge due to its high prevalence and mortality as well as its resistance to available treatments [1,2]. With substantial economic growth and changes in dietary habits, the incidence and mortality rates of CRC have been increasing [3]. In the current clinical paradigm, the management of primary and metastatic CRC predominantly involves surgical resection complemented by adjuvant chemotherapy [4]. However, because of the complexity of the pathogenesis, a substantial number of CRC patients face tumor recurrence due to drug resistance and do not respond to current immune checkpoint therapies [5]. Therefore, it is crucial to identify new potential cancer biomarkers and develop improved therapeutic strategies for CRC.

The epidermal growth factor receptor/mitogen-activated protein kinase (EGFR/MAPK) signaling pathway has an important impact on various cellular processes, encompassing proliferation, differentiation, migration, senescence, and apoptosis [6], and plays a crucial role in the development of multiple cancer hallmarks [7]. Mechanistically, it influences tumor development and progression through a variety of downstream effectors [8,9]. Overactivation of this pathway is extensively documented to correlate with clinical staging and poor patient prognosis in various cancer types, including CRC [10–12]. Cetuximab, an inhibitor of EGFR, has been approved by the US Food and Drug Administration for the treatment of CRC with wild-type Kirsten rat sarcoma viral oncogene homolog (KRAS) and v-Raf murine sarcoma viral oncogene homolog B1 (BRAF) genotypes [13]. However, KRAS and BRAF mutations are prevalent in CRC, leading to the aberrant activation of the MAPK pathway and rendering approximately 60% of CRC patients unresponsive to cetuximab therapy [14]. Therefore, identifying the downstream target genes of the MAPK pathway and elucidating their mechanisms of action are crucial for developing novel therapeutic strategies that target this signaling cascade in CRC.

Noncoding RNAs have emerged as one of the most cutting-edge areas in cancer research, playing critical roles in the regulation of gene expression and cellular processes. Over the past decade, extensive research has revealed that noncoding RNAs, such as microRNAs [15], long noncoding RNAs (lncRNAs) [16,17], circular RNAs [18], and transfer RNA-derived small RNAs [19], are deeply implicated in the hallmarks of cancer. lncRNAs are characterized by a length of over 200 nucleotides and a lack of protein-coding capacity [20]. They exert their functions by interacting with proteins, DNA, or RNA molecules, acting as decoys, scaffolds, or guides, and affecting protein–protein or protein–nucleotide interactions [21–23], thereby regulating targets at transcriptional, translational, or posttranslational levels [24–27]. For example, a hypoxia-induced lncRNA, LVBU (lncRNA regulation via BCL6/urea cycle), functions as a competing endogenous RNA by binding to miR-10a/miR-34c, thereby protecting B-cell lymphoma 6 (BCL6) from degradation mediated by these microRNAs. This protection allows BCL6 to antagonize p53-driven repression of genes involved in the urea cycle/polyamine synthesis [28]. Furthermore, AKT-induced lncRNA VAL (vimentin-associated lncRNA, LINC01546) competes with the E3 ligase tripartite motif containing 16 (Trim16) for binding to vimentin, thereby inhibiting the ubiquitination and degradation of vimentin protein at the posttranslational level, which in turn promotes the metastasis of lung adenocarcinoma [26]. However, it is not clear how many lncRNAs can be regulated by EGFR/MAPK signaling and, if any, what molecular cancer characteristics of these lncRNAs can contribute to EGFR/MAPK-mediated CRC promotion.

Here, we profiled global lncRNA expression upregulated by EGFR/MAPK signaling and identified lncRNA ESSENCE (EGF [epidermal growth factor] Signal Sensing CAD’s Effect; ENST00000415336) induced by the EGF-regulated transcription factor early growth response factor 1 (EGR1). We demonstrate that ESSENCE overexpression correlates with poor prognosis of CRC and is a potent protumorigenic molecule. Mechanistically, ESSENCE increases carbamoyl-phosphate synthetase 2, aspartate transcarbamylase, and dihydroorotase (CAD) stability via preventing E3 ligase KEAP1 (Kelch-like erythroid cell-derived protein with CNC homology-associated protein 1)-mediated CAD ubiquitination and proteasome degradation, thereby promoting CAD-regulated ferroptosis defense and subsequent tumor progression. Importantly, the mitogen-activated extracellular signal-regulated kinase (MEK) inhibitor selumetinib and the ferroptosis inducer sulfasalazine effectively suppress ESSENCE-high CRC in a patient-derived xenograft (PDX) model. Thus, our studies uncover ESSENCE as an important cancer biomarker upregulated by the EGFR/MAPK/EGR1 pathway and also provide insight into CRC diagnosis and treatment.

## Results

### EGFR/MAPK-upregulated lncRNA ESSENCE is positively correlated with poor prognosis in CRC

**Fig. 1.**
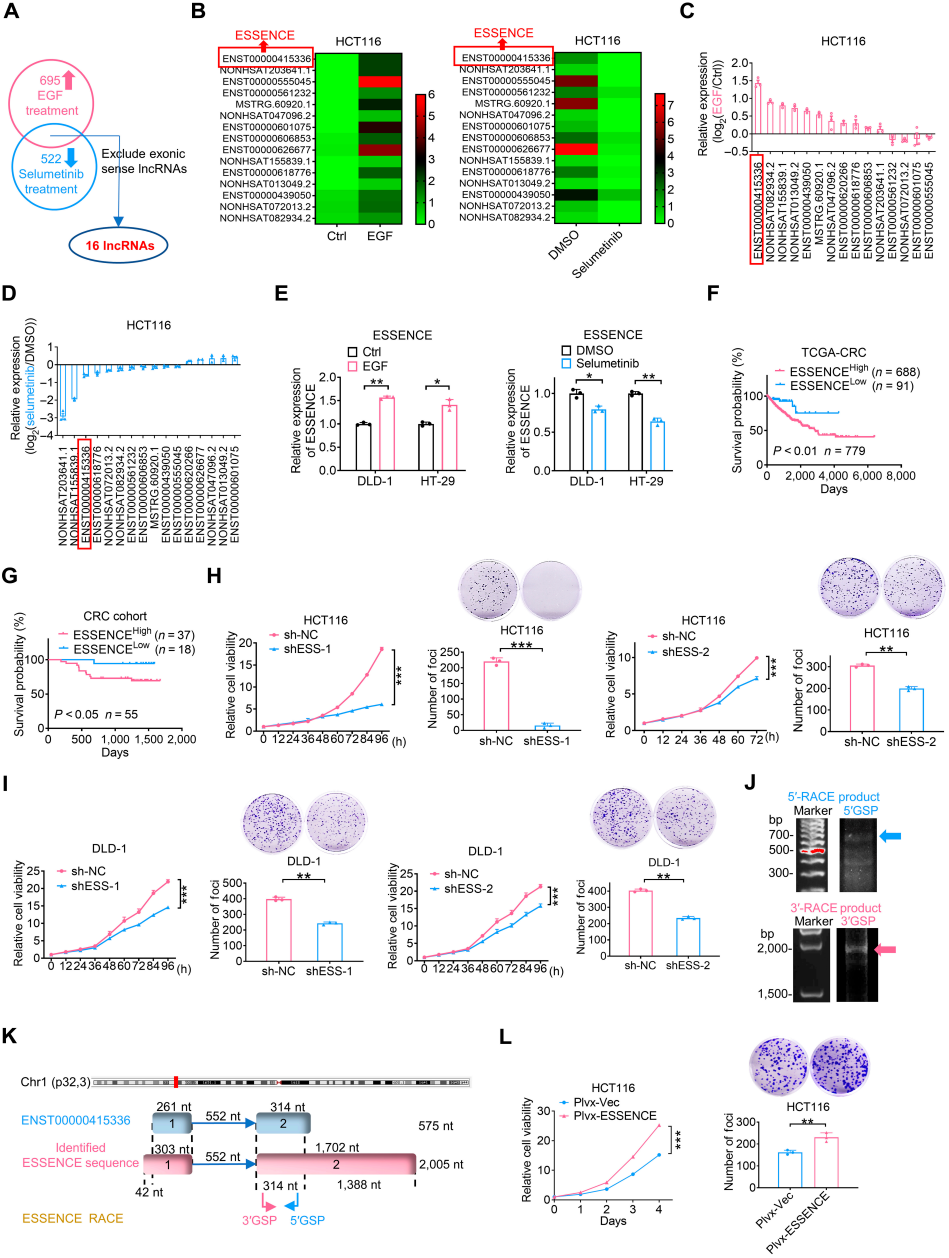
The epidermal growth factor receptor/mitogen-activated protein kinase (EGFR/MAPK)-upregulated long noncoding RNA (lncRNA) ESSENCE (EGF [epidermal growth factor] Signal Sensing CAD’s Effect) is positively correlated with poor prognosis in colorectal cancer (CRC). (A) Overlapping lncRNAs upregulated under EGF treatment and down-regulated under selumetinib treatment by high-throughput sequencing results. (B) The heat map illustrates lncRNAs that have a 2-fold change under EGF or selumetinib treatment. Each row in the heat map corresponds to a distinct lncRNA, and the fold change values have been transformed using a base-2 logarithm. (C and D) The expression of overlapping lncRNAs was detected by quantitative real-time polymerase chain reaction (qRT-PCR) under EGF or selumetinib treatment in HCT116 cells (ENST00000626677 was excluded in (C) due to its cycle threshold (Ct) value >35). (E) Upregulation (down-regulation) of ESSENCE was observed under EGF (selumetinib) treatment in DLD-1 and HT-29 cells through qRT-PCR. (F and G) Kaplan–Meier overall survival (OS) curves were generated based on ESSENCE expression in colon cancer tissues from The Cancer Genome Atlas (TCGA) database (Colon Adenocarcinoma [COAD]) and our CRC cohort. A receiver operating characteristic (ROC) curve was used to define the high- and low-expression groups, and log-rank analysis was used to test for significance. (H and I) Proliferation rate and foci formation of HCT116 and DLD-1 cells were assessed following ESSENCE knockdown (doxycycline induced). (J) Agarose gel electrophoresis was performed on PCR products obtained from 5′-rapid amplification of cDNA (complementary DNA) ends (RACE) and 3′-RACE analyses of ESSENCE (5′GSP represents 5′-RACE gene-specific primer; 3′GSP represents 3′-RACE gene-specific primer). (K) The schematic illustrates the ESSENCE genome sequence, encompassing both exons and introns (length), along with the locations of RACE primer sites. (L) The proliferation rate and foci formation of HCT116 were assessed after stable overexpression of ESSENCE. Data are presented as mean ± SD. *n* = 3 per group. *P < 0.05; **P < 0.01; ***P < 0.001; ns, not significant. Ctrl, control; DMSO, dimethyl sulfoxide; sh-NC, short hairpin RNA-negative control; shESS-1, short hairpin RNA 1-ESSENCE; Plvx-Vec, Plvx-Vector.

EGFR/MAPK signaling exhibits marked activation in cancer [10,29]. In this study, we aimed to investigate how the EGFR/MAPK signaling pathway activates lncRNAs and to examine their potential functions and mechanisms that drive CRC progression [28]. We evaluated the transcriptional expression of lncRNAs in HCT116 cells following treatment with either EGF or selumetinib. Under EGF treatment, 695 lncRNAs exhibited upregulation, whereas under selumetinib treatment, 522 lncRNAs showed down-regulation (Fig. 1A). Overlap of the lncRNAs from these 2 groups, excluding exonic sense lncRNAs, revealed 16 candidate lncRNAs (Fig. 1B). Quantitative real-time polymerase chain reaction (qRT-PCR) analysis showed that there were significant changes in the expression of lncRNA ENST00000415336 (ESSENCE) among them. It ranked as the top 1 upregulated lncRNA following EGF treatment (Fig. 1C) and the top 3 down-regulated lncRNA after selumetinib treatment in HCT116 cells (Fig. 1D). Congruently, we confirmed the upregulation of ESSENCE after EGF treatment and down-regulation after selumetinib treatment in 2 additional CRC cell lines, DLD-1 and HT-29 (Fig. 1E). By conducting bioinformatic analysis on data from The Cancer Genome Atlas (TCGA) and Gene Expression Omnibus (GEO) databases, we observed upregulation of ESSENCE in CRC and adenoma tissues (Fig. S1A and B). Additionally, we evaluated the expression of ESSENCE in 60 patients with CRC. qRT-PCR analysis demonstrated that most of the samples exhibited a significant upregulation of ESSENCE in cancer tissues compared to adjacent nontumor mucosa (Fig. S1C). By contrast, as another potential candidate, lncRNA NONHSAT155839.1 demonstrated down-regulation in most of cancer tissues in CRC cohort (Fig. S1D). Meanwhile, compared to normal epithelial cells (NCM460), most CRC cell lines exhibited elevated levels of ESSENCE expression (Fig. S1E). Furthermore, through Kaplan–Meier analysis, we observed that CRC patients with high ESSENCE expression exhibited poorer overall survival in comparison to those with lower ESSENCE expression in both the TCGA database and CRC cohort (Fig. 1F and G). Hence, we demonstrate that ESSENCE expression is increased in response to EGF stimulation and is correlated with poor prognosis in CRC.

Given ESSENCE’s elevated expression under EGF treatment and its abnormal upregulation in CRC, we proceeded to investigate its biological functions. By introducing short hairpin RNA (shRNA)-mediated ESSENCE knockdown (doxycycline [Dox] induced) in HCT116 and DLD-1 cells and validating the knockdown efficiency through qRT-PCR (Fig. S2A), we observed a significant inhibition of CRC cell proliferation and foci formation (Fig. 1H and I). Furthermore, through the 5′- and 3′-rapid amplification of cDNA (complementary DNA) ends (RACE) assay (Fig. 1J and Fig. S2B), we found that lncRNA ESSENCE is a 2,005-nt transcript comprising 2 exons and 1 intron (Fig. 1K and Fig. S2C). We then constructed ESSENCE overexpression plasmids and validated the overexpression efficiency through qRT-PCR (Fig. S2D and E). As a result, we observed a significant increase in cell proliferation and foci formation after transient or stable overexpression of ESSENCE in CRC cells (Fig. 1L and Fig. S2F and G).

To further characterize ESSENCE, we employed the RNAfold web server (http://rna.tbi.univie.ac.at//cgi-bin/RNAWebSuite/RNAfold.cgi/) to predict the secondary structure of ESSENCE (Fig. S2H). We then assessed the coding probability of ESSENCE utilizing the Coding Potential Assessment Tool (CPAT) (http://lilab.research.bcm.edu/cpat) and the Coding Potential Calculator 2 (CPC2) (http://cpc2.gao-lab.org/). CPAT predicted a coding probability of 0.00998, while CPC2 assigned a score of 0.10830, both reinforcing the conclusion that ESSENCE lacks protein-coding potential (Fig. S2I). Together, these results suggest that ESSENCE plays an oncogenic role in regulating CRC cell growth.

### EGF-upregulated lncRNA ESSENCE expression is mediated by the EGF downstream transcription factor EGR1

**Fig. 2.**
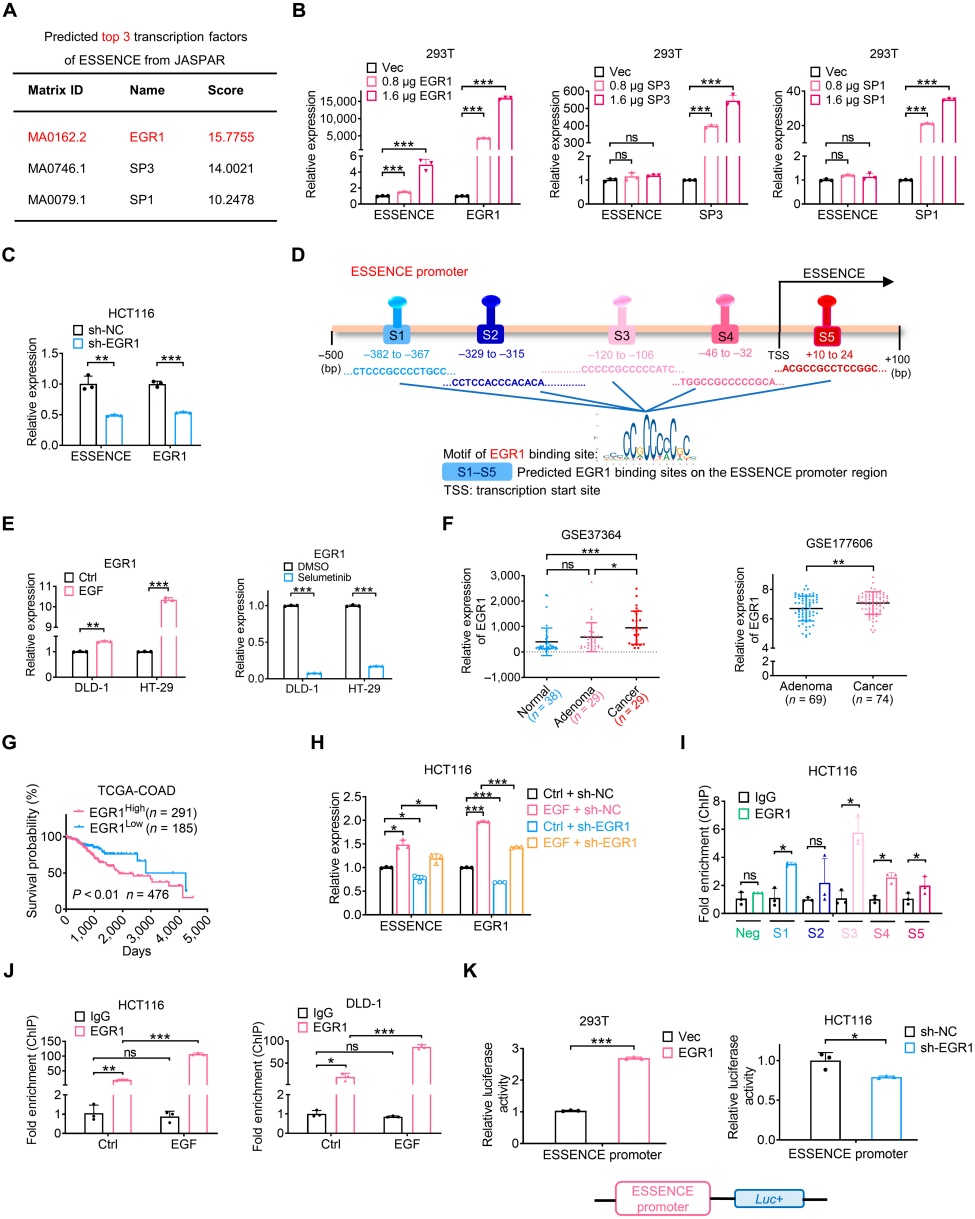
EGF-upregulated lncRNA ESSENCE expression is mediated by the EGF downstream transcription factor early growth response factor 1 (EGR1). (A) The top 3 potential transcription factors of ESSENCE were predicted by the website JASPAR. (B) The expression of ESSENCE was determined by qRT-PCR following overexpression of EGR1, specificity protein 3 (SP3), and specificity protein 1 (SP1) in 293T cells. (C) The expression of ESSENCE and EGR1 was determined by qRT-PCR following EGR1 knockdown in HCT116 cells. (D) The predicted binding sites (S1 to S5) of EGR1 at the promoter region (from −2,000 to +100 bp) of ESSENCE are shown. (E) The expression of EGR1 was detected by qRT-PCR under EGF (left) or selumetinib (right) treatment in DLD-1 and HT-29 cells. (F) Expression levels of EGR1 in normal mucosa, adenoma, and CRC tumor tissues from the GSE37364 and GSE177606 databases. (G) Kaplan–Meier overall OS curves were generated based on EGR1 expression in colon cancer tissues from TCGA database (COAD), with significance testing carried out using log-rank analysis.(H) The expression of ESSENCE and EGR1 was determined by qRT-PCR following EGR1 knockdown in HCT116 cells under EGF treatment. (I) Different binding sites of EGR1 on ESSENCE promoter was confirmed by chromatin immunoprecipitation (ChIP) assays in HCT116 cells. (J) The binding of EGR1 to the ESSENCE promoter was evaluated by ChIP assay after EGF treatment. (K) The luciferase activities of the ESSENCE promoter were determined by a dual-luciferase reporter assay following EGR1 overexpression or knockdown. Data are presented as mean ± SD. *n* = 3 per group. *P < 0.05; **P < 0.01; ***P < 0.001; ns, not significant. sh-NC, short hairpin RNA-negative control; sh-EGR1, short hairpin RNA-EGR1; IgG, immunoglobulin G.

To gain insights into the mechanism responsible for the upregulation of ESSENCE during EGF treatment, we conducted a bioinformatic analysis of the ESSENCE promoter region utilizing JASPAR (https://jaspar.genereg.net/). EGR1, specificity protein 3 (SP3), and specificity protein 1 (SP1) were the top 3 predicted transcription factors for ESSENCE, with a score of 15.7755 assigned to EGR1 (Fig. 2A). We then constructed overexpression plasmids for EGR1, SP3, and SP1. Our qRT-PCR results revealed that ESSENCE was upregulated after EGR1 overexpression, whereas overexpression of SP3 and SP1 had little impact on ESSENCE expression (Fig. 2B). In contrast, silencing EGR1 suppressed the expression of ESSENCE (Fig. 2C). Five potential binding sites of EGR1 were found in the promoter region of ESSENCE (S1 to S5) based on JASPAR (Fig. 2D). EGF triggers a cascade of events, starting with the activation of EGFR and subsequently leading to the activation of rat sarcoma (RAS), RAF, MEK, and extracellular signal-regulated kinase (ERK), ultimately resulting in the upregulation of EGR1 [30]. Our results confirmed a significant increase in EGR1 levels following EGF treatment, while EGR1 levels decreased after selumetinib treatment in both DLD-1 and HT-29 cells (Fig. 2E). By conducting bioinformatic analysis on data from GEO databases, we observed upregulation of EGR1 in CRC tissues compared to that in normal mucosa and adenoma (Fig. 2F). Furthermore, through Kaplan–Meier analysis, we found that CRC patients with high EGR1 expression exhibited poorer overall survival in comparison to those with lower EGR1 expression in the TCGA database (Fig. 2G). Additionally, we observed that silencing EGR1 significantly attenuated EGF-induced upregulation of ESSENCE expression (Fig. 2H). Chromatin immunoprecipitation (ChIP) assays provided confirmation of EGR1 binding to the ESSENCE promoter at sites S1, S3, S4, and S5 (Fig. 2I). Notably, the binding efficiency of EGR1 increased following EGF treatment in both HCT116 and DLD-1 cells (Fig. 2J). By constructing the ESSENCE promoter region into a luciferase reporter, we observed that EGR1 overexpression led to an increase in ESSENCE luciferase activity, whereas silencing EGR1 resulted in decreased luciferase activity (Fig. 2K). These findings reveal that the upregulation of ESSENCE induced by EGF treatment is mediated by the binding of EGR1 to the ESSENCE promoter in CRC cells.

### ESSENCE interacts with CAD and regulates CAD’s steady-state expression

**Fig. 3.**
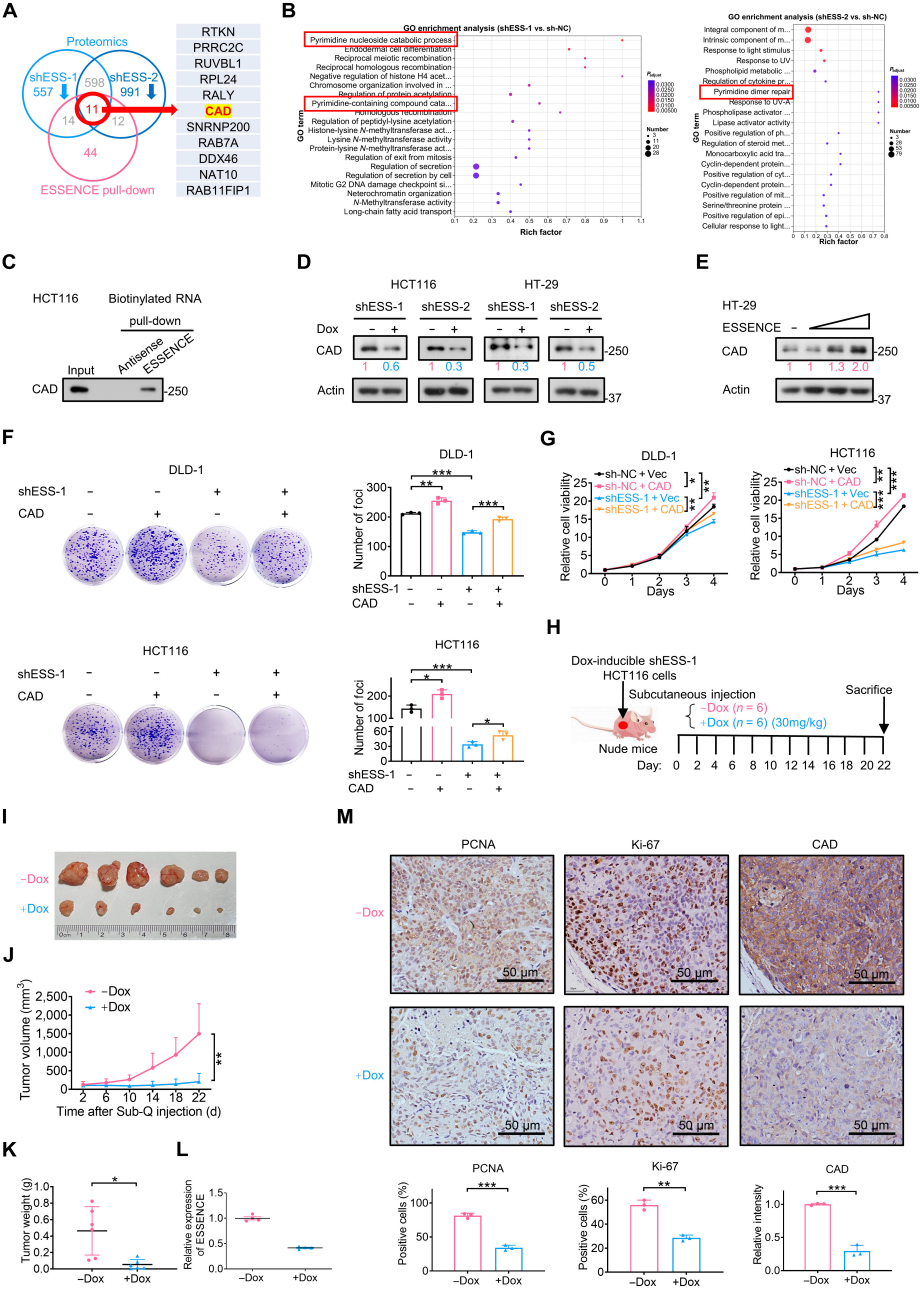
ESSENCE interacts with carbamoyl-phosphate synthetase 2, aspartate transcarbamylase, and dihydroorotase (CAD) and regulates CAD’s steady-state expression. (A) List of overlapping proteins that are both regulated and associated with ESSENCE. (B) Gene Ontology (GO) enrichment analysis of ESSENCE-regulated proteins based on proteomics. (C) Representative immunoblot results of CAD pulled down from HCT116 cell lysates by ESSENCE antisense (negative control) or ESSENCE. (D and E) Representative immunoblot results of CAD steady-state expression following ESSENCE knockdown or overexpression in HCT116 or HT-29 cells. Short hairpin RNA (shRNA)-mediated ESSENCE knockdown (doxycycline induced) was introduced into HCT116 and HT-29 cells. (F and G) The foci formation and proliferation rate of DLD-1 and HCT116 cells were assessed following the indicated treatment. (H) Schematic depicting the xenograft mouse model. (−Dox, phosphate-buffered saline (PBS); +Dox, shESS-1). Dox, doxycycline. (I) Representative images of xenograft tumors from mice. (J and K) Tumor growth curves and tumor weight are shown. (L) The relative ESSENCE expression levels of xenograft tumors from mice were measured by qRT-PCR. (M) Representative immunohistochemical (IHC) staining results of proliferating cell nuclear antigen (PCNA), Ki-67, and CAD in tumor tissues obtained from (I) (upper). Quantifications of IHC staining were analyzed by ImageJ and are shown as bar graphs (lower). Data are presented as mean ± SD. *n* = 3 per group. *P < 0.05; **P < 0.01; ***P < 0.001; ns, not significant. Sub-Q, subcutaneous.

We next proceeded to investigate the underlying molecular mechanism of ESSENCE functions. Given that other lncRNAs have been observed to interact with and regulate the functions of proteins [31,32], we conducted an RNA–protein pull-down assay to identify potential proteins interacted with ESSENCE (Fig. S3A). Overlap of the candidate proteins from pull-down mass spectrometry and proteomics (proteins that are down-regulated by ESSENCE knockdown) unraveled 11 proteins, including the multifunctional enzyme CAD (Fig. 3A). Also, Gene Ontology enrichment analysis of ESSENCE-regulated proteins (proteomics) revealed that the pyrimidine nucleoside catabolic process, pyrimidine-containing compound catabolic process, and pyrimidine dimer repair process were enriched (Fig. 3B). CAD plays a pivotal role in catalyzing the first 3 steps of de novo pyrimidine synthesis [33]. In mammals, the activation of de novo pyrimidine synthesis is induced by growth factors [34]. Nevertheless, the precise mechanisms of its regulation by growth cues and lncRNA remain not well characterized. Therefore, we hypothesized that CAD might be a potential effector influenced by ESSENCE. Bioinformatic analysis revealed that CAD exhibited significantly higher protein levels in colon cancer tissues compared to adjacent nontumor mucosa, as observed in the Clinical Proteomic Tumor Analysis Consortium database (Fig. S3B). Congruently, we observed an upregulation of CAD in CRC tissues (Fig. S3C), and a high level of CAD was found to correlate with poor survival in colon cancer based on the TCGA database (Fig. S3D). We subsequently performed an RNA pull-down assay, complemented by immunoblotting, and confirmed that CAD was precipitated by ESSENCE RNA but not by antisense ESSENCE RNA (Fig. 3C). Following this, we proceeded to explore the regulatory mechanism of ESSENCE on CAD. Immunoblotting analysis revealed that knocking down ESSENCE led to a decrease in the steady-state expression of CAD in HCT116 and HT-29 (Fig. 3D). Conversely, overexpression of ESSENCE markedly elevated CAD protein levels in a dose-dependent manner (Fig. 3E). More importantly, functional studies revealed that overexpression of CAD alleviated the inhibitory effects on cell viability and foci formation induced by ESSENCE knockdown (Fig. 3F and G). Collectively, these findings suggest that ESSENCE interacts with CAD and regulates CAD’s steady-state expression.

To investigate the in vivo impact of ESSENCE on tumorigenesis, we established a CRC xenograft mouse model by subcutaneously implanting cells with Dox-inducible stable ESSENCE knockdown (Fig. 3H). The mice harboring ESSENCE knockdown cells (+Dox) displayed reduced tumor growth compared to the control group (−Dox) (Fig. 3I to K). qRT-PCR analysis of tumor tissues derived from the mouse xenografts confirmed the effectiveness of ESSENCE knockdown (Fig. 3L). Notably, ESSENCE knockdown tumors also exhibited decreased expression of proliferating cell nuclear antigen (PCNA) and Ki-67 (proliferation markers) and CAD, as demonstrated by immunohistochemistry staining (Fig. 3M). Together, these data indicate that CAD is a functional target of ESSENCE and may be involved in ESSENCE-mediated tumorigenesis of CRC.

### ESSENCE inhibits E3 ubiquitin ligase KEAP1-mediated degradation of CAD

We observed that knockdown ESSENCE in CRC cells resulted in a marked reduction in the steady-state expression of CAD protein levels, while ESSENCE overexpression increased CAD protein levels. A qRT-PCR assay indicated that either overexpression or knockdown of ESSENCE had no impacts on the messenger RNA (mRNA) expression of CAD (Fig. S3E and F), suggesting that ESSENCE regulates CAD at the posttranscriptional level.

**Fig. 4.**
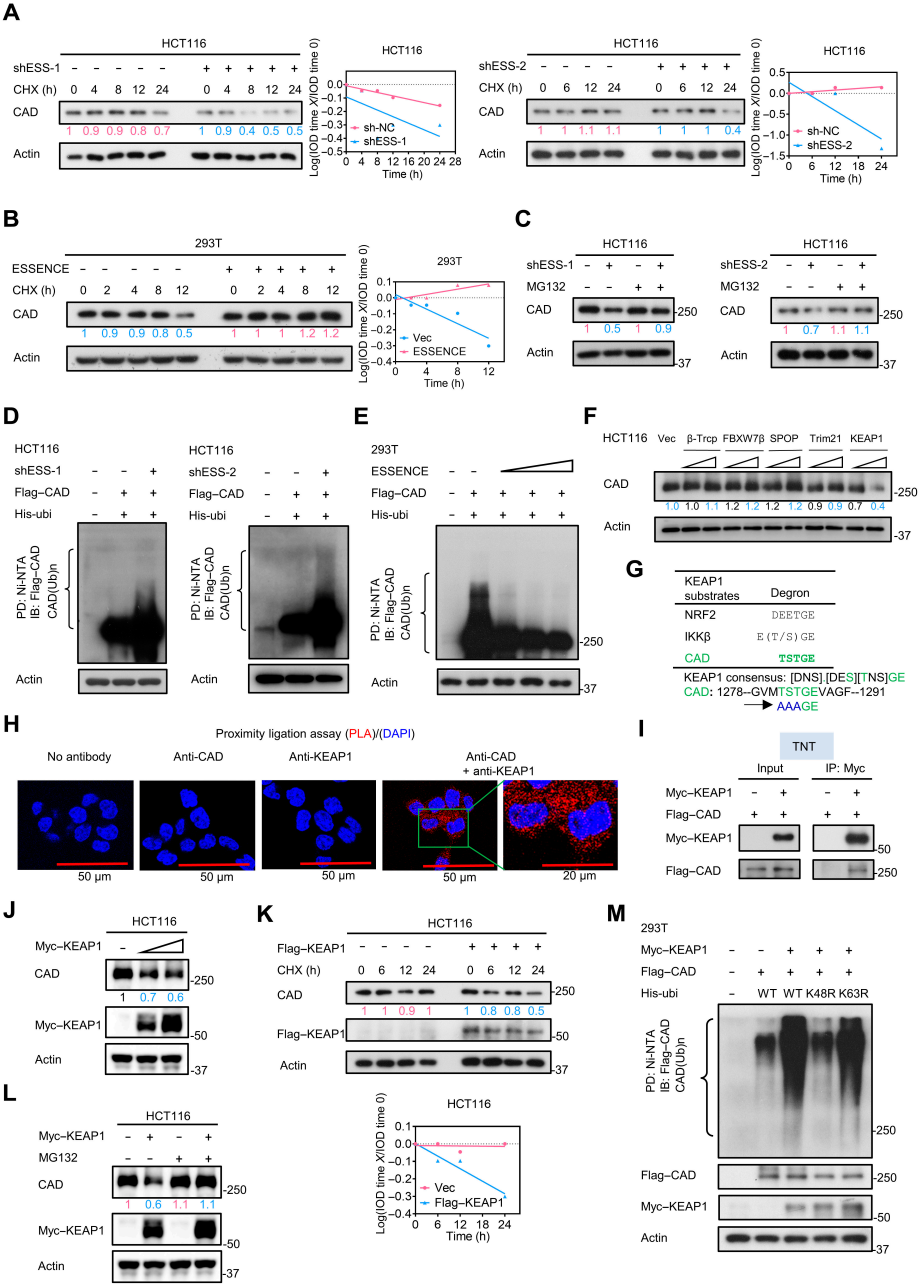
ESSENCE inhibits E3 ubiquitin ligase KEAP1 (Kelch-like erythroid cell-derived protein with CNC homology-associated protein 1)-mediated degradation of CAD. (A and B) HCT116 or 293T cells were treated with cycloheximide (CHX) (160 μg ml^−1^) for the indicated times following ESSENCE knockdown or overexpression. Lysates were immunoblotted with indicated antibodies. Quantification of CAD turnover rate was analyzed by ImageJ. (C) HCT116 cells were treated with or without proteasome inhibitor MG132 following ESSENCE knockdown. Lysates were immunoblotted with indicated antibodies. (D and E) Cells transfected with the indicated plasmids were treated with 50 μM MG132 for 6 h before harvesting. Cell lysates were pulled down (PD) with nickel beads (nickel–nitrilotriacetic acid [Ni-NTA]) and immunoblotted (IB) with indicated antibodies. (F) Representative immunoblot results of CAD steady-state expression following overexpression of indicated E3 ligases in HCT116 cells. (G) Sequence alignment of the putative KEAP1-recognized degron on CAD. [DNS] refers to a group of amino acids (Asp, Asn, Ser) that are all allowed; [DES] refers to a group of amino acids (Asp, Glu, Ser) that are all allowed; [TNS] refers to a group of amino acids (Thr, Asn, Ser) that are all allowed. (H) Representative image results of proximity ligation assay (PLA) using antibodies against CAD and KEAP1. The red signals indicate interactions between CAD and KEAP1. 4′,6-diamidino-2-phenylindole (DAPI) was used to stain the cell nuclei (blue signals). (I) Anti-Myc magnetic beads were incubated with Myc–KEAP1 and Flag–CAD proteins, which were obtained from TNT Quick Coupled Transcription/Translation systems. Immunoblotting was conducted using the indicated antibodies. (J) Representative immunoblot of CAD steady-state expression following KEAP1 overexpression in HCT116. (K) HCT116 cells were treated with cycloheximide (CHX) (160 μg ml^−1^) for the indicated times following KEAP1 overexpression. Lysates were immunoblotted with indicated antibodies. Quantification of CAD turnover rate was analyzed by ImageJ. (L) HCT116 cells were treated with or without proteasome inhibitor MG132 following KEAP1 overexpression. Lysates were immunoblotted with indicated antibodies. (M) 293T cells transfected with the indicated plasmids were treated with 50 μM MG132 for 6 h before harvesting. Cell lysates were pulled down (PD) with nickel beads (Ni-NTA) and immunoblotted (IB) with indicated antibodies. IOD, integrated optical density; His-ubi, His-ubiquitin; CAD (Ub)n, CAD ubiquitination; β-Trcp, beta-transducin repeats-containing proteins; FBXW7β, F-box and WD repeat domain containing 7 beta; SPOP, speckle-type POZ protein; Trim21, tripartite motif protein 21; NRF2, nuclear factor erythroid 2-related factor 2; IKKβ, inhibitory kappa B kinase beta; TNT, Transcription and Translation; WT, wild type.

In order to further investigate the mechanism underlying ESSENCE-mediated stabilization of CAD protein levels, we examined the protein stability of CAD under cycloheximide (CHX) treatment. Our results revealed that knockdown of ESSENCE accelerated the turnover rate of CAD (Fig. 4A), whereas ESSENCE overexpression extended the half-life of CAD protein (Fig. 4B). Further, ESSENCE knockdown-mediated down-regulation of CAD can be antagonized by proteasome inhibitor MG132 (Fig. 4C). Consistently, we observed an increase in the ubiquitination level of CAD in response to ESSENCE knockdown (Fig. 4D). By contrast, the ubiquitination level of CAD was diminished under ESSENCE overexpression in a dose-dependent manner (Fig. 4E and Fig. S3G), suggesting that ESSENCE-regulated upregulation of CAD involves a reduced polyubiquitination process. Further, we showed that ESSENCE-mediated ubiquitination of CAD involves the K48 site (Fig. S4A), a common linkage for protein degradation. Collectively, these findings demonstrate that ESSENCE attenuates polyubiquitination-mediated degradation of CAD protein, thereby resulting in an increase in CAD protein levels.

To characterize whether a specific E3 ligase is involved in CAD ubiquitination, we selected tumor suppressor type E3 ubiquitin ligases for screening, such as beta-transducin repeats-containing proteins (β-Trcp), F-box and WD repeat domain containing 7 beta (FBXW7β), speckle-type POZ protein (SPOP), tripartite motif protein 21 (Trim21), and KEAP1 [35–39]. The results revealed that overexpression of KEAP1 significantly reduced the steady-state expression of CAD (Fig. 4F). KEAP1 employs a consensus sequence to recognize its target proteins [40], such as nuclear factor erythroid 2-related factor 2 (NRF2) and inhibitory kappa B kinase beta (Fig. 4G) [39,41]. Subsequently, we scrutinized the CAD peptide sequence and identified the presence of a KEAP1-binding degron (1281 TSTGE 1287) in CAD (Fig. 4G). The 3-dimensional structure of KEAP1-binding degron of CAD was demonstrated using the PyMOL visualization software (Fig. S4B). To demonstrate the interaction between KEAP1 and CAD, exogenous co-immunoprecipitation (Co-IP) was conducted, and the results showed that CAD was co-precipitated with KEAP1 (Fig. S4C). We further documented that KEAP1 interacted with CAD based on in situ proximity ligation assay (PLA) (Fig. 4H). Congruently, we performed an in vitro Transcription and Translation (TNT) assay, complemented by Co-IP, thus verifying the direct binding between KEAP1 and CAD (Fig. 4I). Importantly, overexpression of KEAP1 diminished the steady-state expression of CAD (Fig. 4J and Fig. S4D) and accelerated the turnover rate of CAD in HCT116 and 293T cells (Fig. 4K and Fig. S4E). Also, KEAP1 overexpression-mediated down-regulation of CAD can be antagonized by proteasome inhibitor MG132 (Fig. 4L). Finally, we observed an increase in the ubiquitination level of CAD under KEAP1 overexpression (Fig. S4F). We showed that KEAP1 utilized the wild type and the K63R mutant His-Ubi but not the K48R mutant His-Ubi to induce CAD ubiquitination, suggesting that KEAP1 mediates CAD ubiquitination through a K48 linkage (Fig. 4M), a process typically associated with protein degradation [42]. Taken together, these data demonstrate that KEAP1 is the E3 ligase for CAD, promoting CAD ubiquitination and degradation.

Based on the prediction of the binding site of CAD, we mutated the 1281TST motif into alanine (1281TST → AAA) and found that Co-IP studies demonstrated a significant decrease in the interaction between the CAD (1281TST → AAA) mutant and KEAP1 (Fig. S4G), suggesting that the 1281TST sequence within CAD serves as the crucial binding site for KEAP1. Consistently, the CAD (1281TST → AAA) mutant is resistant to KEAP1-mediated ubiquitination (Fig. S4H). Collectively, these findings indicate that KEAP1 binds CAD through the 1281TST motif on CAD and that this direct binding is critical for KEAP1-mediated CAD ubiquitination and degradation.

### ESSENCE attenuates the interaction between KEAP1 and CAD

**Fig. 5.**
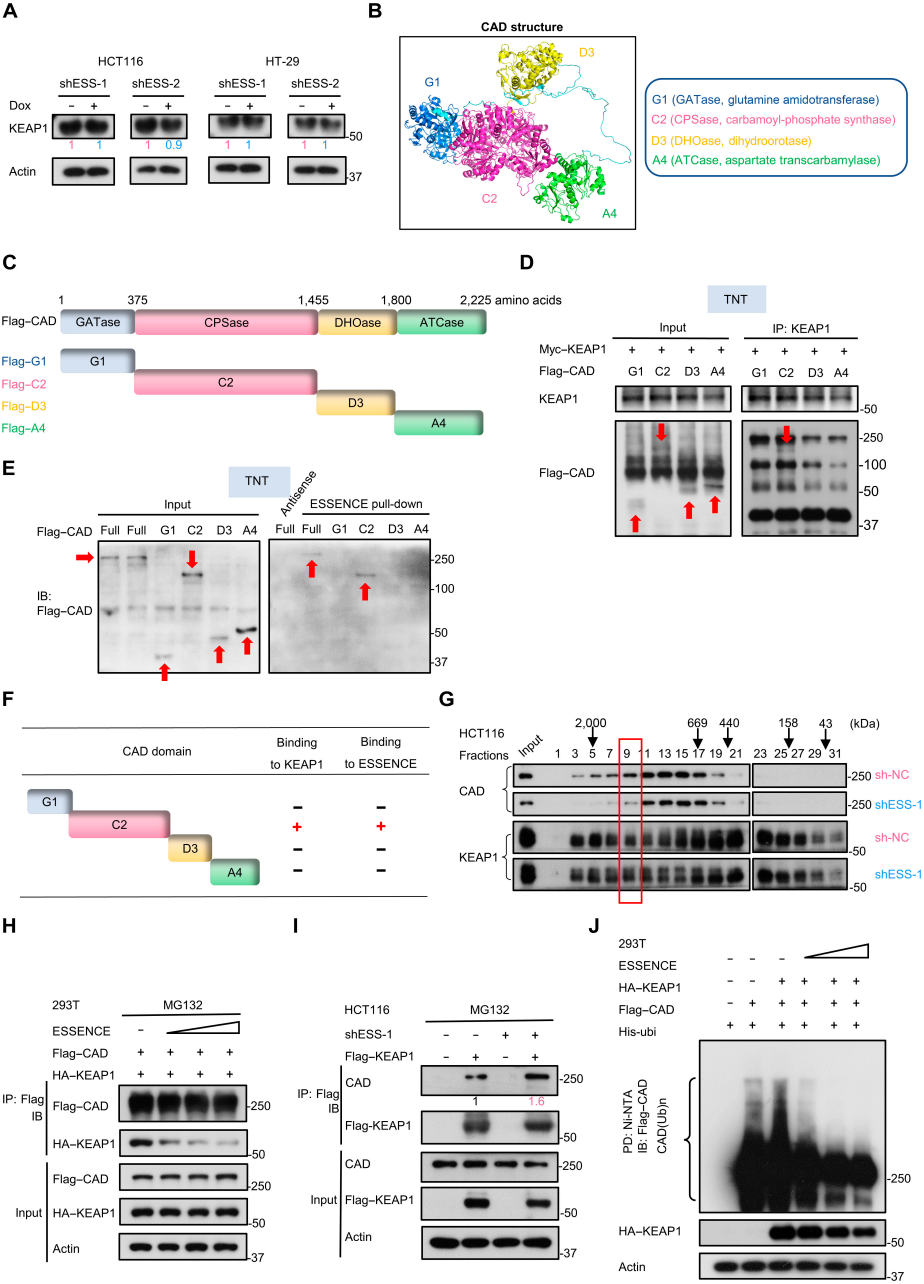
ESSENCE attenuates the interaction between KEAP1 and CAD. (A) Representative immunoblot results of KEAP1 steady-state expression following ESSENCE knockdown (doxycycline induced) in HCT116 and HT-29 cells. (B) Schematic drawing of the secondary structure of CAD (4 domains) predicted with PyMOL. (C) Schematic depiction of wild-type and truncated mutants of CAD. (D) Myc–KEAP1 and Flag–CAD proteins, which were obtained from TNT Quick Coupled Transcription/Translation systems, were pulled down by Protein A/G Agarose beads plus KEAP1 antibody. Immunoblotting was conducted using the indicated antibodies. (E) Streptavidin magnetic beads were incubated with biotinylated ESSENCE (obtained from TranscriptAid T7 High Yield Transcription Kit) and Flag–CAD protein (obtained from TNT Quick Coupled Transcription/Translation systems) mixtures. Immunoblotting was conducted using Flag antibody. (F) Schematic depiction of the conclusions from (D) and (E) results. (G) Analysis of cell lysates from sh-NC and shESS-1 HCT116 cells by gel filtration chromatography. The molecular weight of the eluted fraction is shown above. (H and I) The indicated plasmids were transfected into the cells, followed by immunoprecipitation with M2 beads and immunoblotting with indicated antibodies. (J) 293T cells transfected with the indicated plasmids were treated with 50 μM MG132 for 6 h before harvesting. Cell lysates were pulled down (PD) with nickel beads (Ni-NTA) and immunoblotted (IB) with indicated antibodies. HA-KEAP1, hemagglutinin tag KEAP1.

Subsequently, we investigated how ESSENCE plays a role in KEAP1-mediated CAD degradation. Immunoblotting analysis showed that ESSENCE had no impact on the steady-state expression of KEAP1 protein (Fig. 5A). We then generated the 3-dimensional secondary structure of CAD using the PyMOL visualization software and constructed truncated constructs of CAD domains to perform structure and function studies (Fig. 5B and C). Four domains of CAD were constructed individually, including glutamine amidotransferase (GATase, G1), carbamoyl-phosphate synthase (CPSase, C2), dihydroorotase (DHOase, D3), and aspartate transcarbamoylase (ATCase, A4) (Fig. 5C). We further performed a TNT assay using various truncated constructs of CAD domains, complemented by Co-IP, thus demonstrating that KEAP1 bound to the C2 domain of CAD, where the recognition degron is also located (Fig. 5D). Surprisingly, ESSENCE RNA pull-down assay using 4 TNT truncated constructs of CAD domains indicated that ESSENCE also bound to the C2 domain of CAD (Fig. 5E). These results demonstrate that KEAP1 and ESSENCE bind to the same C2 domain of CAD (Fig. 5F). Based on these findings, we hypothesized that ESSENCE might compete with KEAP1 for binding to CAD, thereby abrogating the interaction between KEAP1 and CAD when overexpressed. Indeed, we observed an increased dynamic complex formation pattern between CAD and KEAP1 when ESSENCE was knocked down as demonstrated by their coelution in a gel filtration experiment (Fig. 5G). Moreover, increasing amounts of ESSENCE resulted in a dose-dependent reduction in the binding between CAD and KEAP1 in 293T cells (Fig. 5H). In contrast, knockdown of ESSENCE led to an increased binding between KEAP1 and CAD (Fig. 5I). Notably, overexpression of ESSENCE could largely mitigate KEAP1-induced polyubiquitination of CAD (Fig. 5J). Together, these findings suggest that ESSENCE competitively binds to CAD, abrogating the interaction of CAD with its E3 ligase KEAP1, thereby disrupting KEAP1-dependent ubiquitination and degradation of CAD.

### ESSENCE mediates ferroptosis defense through the CAD–DHODH axis

**Fig. 6.**
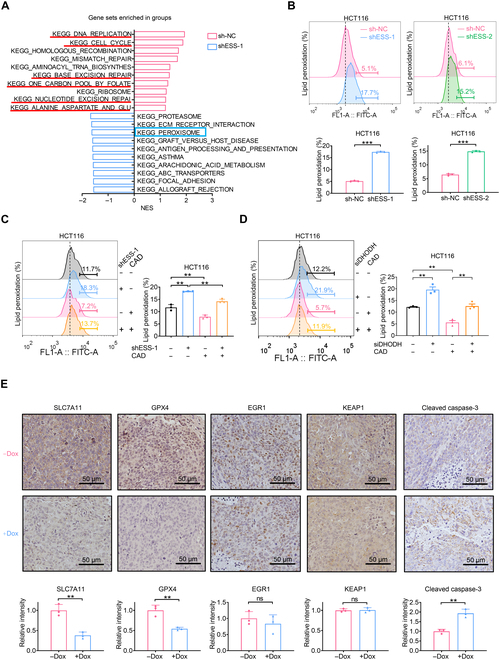
ESSENCE mediates ferroptosis defense through the CAD–dihydroorotate dehydrogenase (DHODH) axis. (A) Gene set enrichment analysis (GSEA) of RNA sequencing (RNA-seq) data showing the top 10 Kyoto Encyclopedia of Genes and Genomes (KEGG) pathways enriched in the sh-NC group and shESS-1 group. The normalized enrichment score (NES) is shown. (B) Levels of lipid peroxidation were measured following ESSENCE knockdown in HCT116 cells. (C) Levels of lipid peroxidation were measured following ESSENCE knockdown and transfection with CAD plasmids in HCT116 cells. (D) Levels of lipid peroxidation were measured following transfection with DHODH small interfering RNA (siRNA) and CAD expression plasmid in HCT116 cells. (E) Representative IHC staining of SLC7A11, glutathione peroxidase 4 (GPX4), EGR1, KEAP1, and cleaved caspase-3 in xenograft tumors from mice (upper). Quantifications of IHC staining were analyzed by ImageJ and are shown as bar graphs (lower). Data are presented as mean ± SD. *n* = 3 per group. *P < 0.05; **P < 0.01; ***P < 0.001; ns, not significant. FL1-A, fluorescence channel 1-area; FITC-A, fluorescein isothiocyanate-area; siDHODH, small interfering RNA of DHODH.

To further explore the detailed mechanisms of ESSENCE involved in CRC progression, we conducted a transcriptome analysis upon silencing ESSENCE in HCT116 cells. Analysis of the RNA sequencing data using gene set enrichment analysis (GSEA) indicated enrichment of numerous pathways related to cell proliferation and metabolic processes. These pathways encompassed DNA replication, cell cycle, base excision repair, one-carbon pool by folate, nucleotide excision repair, and alanine, aspartate, and glutamate metabolism, all of which exhibited pronounced enrichment in the ESSENCE high-expression group (Fig. 6A). Additionally, GSEA also identified that the peroxisome pathway was enriched in the ESSENCE knockdown group (Fig. 6A), which is essential for driving ferroptosis [43]. Since dihydroorotate dehydrogenase (DHODH), a critical downstream enzyme of CAD [44], functions concurrently with mitochondrial glutathione peroxidase 4 (GPX4) to inhibit ferroptosis by converting ubiquinone to ubiquinol [45], we hypothesized that ESSENCE might regulate ferroptosis through the CAD–DHODH axis. To test this hypothesis, we detected the levels of lipid peroxidation following ESSENCE knockdown. As anticipated, ESSENCE knockdown induced lipid peroxidation and ferroptosis in HCT116 and DLD-1 cells (Fig. 6B and Fig. S5A). In parallel, knockdown of CAD using small interfering RNAs also showed elevated levels of lipid peroxidation in DLD-1 cells (Fig. S5B). Notably, the elevated levels of lipid peroxidation in ESSENCE-knockdown cells could be reversed by CAD overexpression in HCT116 and DLD-1 cells (Fig. 6C and Fig. S5C), suggesting that ESSENCE’s impact on ferroptosis is mediated through CAD. As expected, the reduction of lipid peroxidation observed in CAD-overexpressing cells could be restored following DHODH knockdown (Fig. 6D), indicating that the inhibition of ferroptosis by CAD overexpression is indeed mediated through DHODH.

Notably, in ESSENCE knockdown tumors (CRC xenograft mouse model in Fig. 3H), there was a concurrent decrease in the expression of GPX4 and SLC7A11 as demonstrated by immunohistochemistry staining (Fig. 6E). The SLC7A11–GPX4 axis is widely recognized as the most crucial system in ferroptosis defense [46]. Therefore, ESSENCE’s impact on ferroptosis defense is manifested in the CAD–DHODH axis as well as SLC7A11 and GPX4 expression.

### Combination treatment of the MEK inhibitor selumetinib and the ferroptosis inducer sulfasalazine inhibited tumor growth in ESSENCE-high human CRC PDX models

**Fig. 7.**
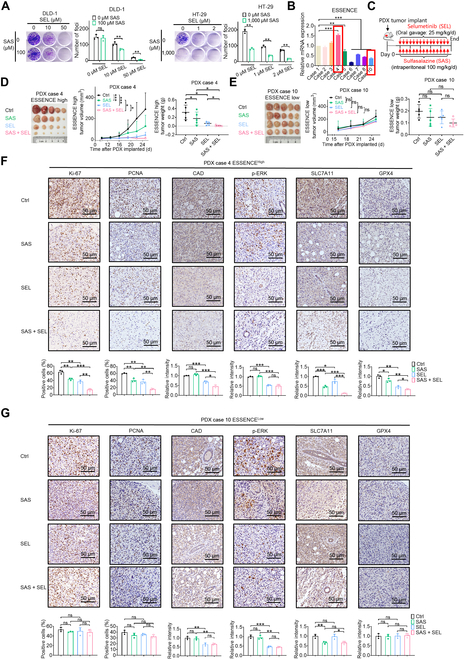
Combination treatment of the mitogen-activated extracellular signal-regulated kinase (MEK) inhibitor selumetinib and ferroptosis inducer sulfasalazine inhibited tumor growth in ESSENCE-high human CRC a patient-derived xenograft (PDX) models. (A) Foci formation of DLD-1 and HT-29 cells was conducted following treatment with selumetinib and sulfasalazine at different doses and combinations. Quantifications of foci formation are shown as bar graphs. (B) Relative ESSENCE expression levels of indicated PDX tumor tissues by qRT-PCR. (C) Schematic depiction of PDX tumors’ treatment schedule in immunocompromised mice. (D and E) Representative images of PDX tumors, tumor growth curves, and tumor weight of mice engrafted with indicated CRC PDXs and treatments are shown (*n* = 5). (F and G) Representative IHC staining results of Ki-67, PCNA, CAD, phosphorylated extracellular signal-regulated kinase (p-ERK), SLC7A11, and GPX4 in tumor tissues obtained from (D) and (E). Quantifications of IHC staining were analyzed by ImageJ and are shown as bar graphs (lower). Data are presented as mean ± SD. *P < 0.05; **P < 0.01; ***P < 0.001; ns, not significant. mRNA, messenger RNA.

To explore the potential of targeting the ESSENCE–CAD–ferroptosis axis in restraining CRC development, we conducted cell growth experiments using both selumetinib (MEK inhibitor, SEL) and sulfasalazine (ferroptosis inducer, SAS) to impede CRC cells’ growth. Notably, the combined treatment of selumetinib and sulfasalazine exhibited greater efficacy in suppressing foci formation of CRC cells when compared with either selumetinib or sulfasalazine alone (Fig. 7A). To evaluate the applicability of our discoveries to human CRC, we established PDX models [36,47] to assess the efficacy of combined treatment in inhibiting CRC tumor growth across 2 sets of CRC PDXs categorized as ESSENCE high or ESSENCE low, with ESSENCE expression levels confirmed by qRT-PCR (Fig. 7B and C). Notably, the combination of selumetinib and sulfasalazine demonstrated superior efficacy in suppressing tumor growth compared to using selumetinib or sulfasalazine alone in the ESSENCE-high PDX tumors (case 4) (Fig. 7D). In contrast, the combination treatment had less dramatic impact on ESSENCE-low PDX tumors (case 10) in terms of both tumor volume and weight (Fig. 7E). As expected, administration of the selumetinib and sulfasalazine combination in ESSENCE-high tumors significantly reduced Ki-67 and PCNA expression, whereas it had a minimal impact in the ESSENCE-low tumors (Fig. 7F and G). Combination treatment in the ESSENCE-high tumors significantly reduced CAD expression compared to using selumetinib or sulfasalazine alone, whereas it had no synergistic impact in the ESSENCE-low tumors (Fig. 7F and G). Congruently, phosphorylated extracellular signal-regulated kinase (p-ERK) is down-regulated in the selumetinib treatment group, and SLC7A11 is down-regulated in the sulfasalazine treatment group, confirming drug efficiency (Fig. 7F and G). As observed in the mouse CRC xenograft model, ESSENCE-high PDX tumors had reduced SLC7A11 and GPX4 expression in the selumetinib treatment group since ERK–ESSENCE–ferroptosis defense signaling is intercepted (Fig. 7F). Taken together, these findings suggest that ESSENCE is an actionable biomarker and that EGF signaling inhibitor selumetinib and ferroptosis inducer sulfasalazine combination treatment may hold promise for the treatment of CRC patients with high ESSENCE expression.

## Discussion

**Fig. 8.**
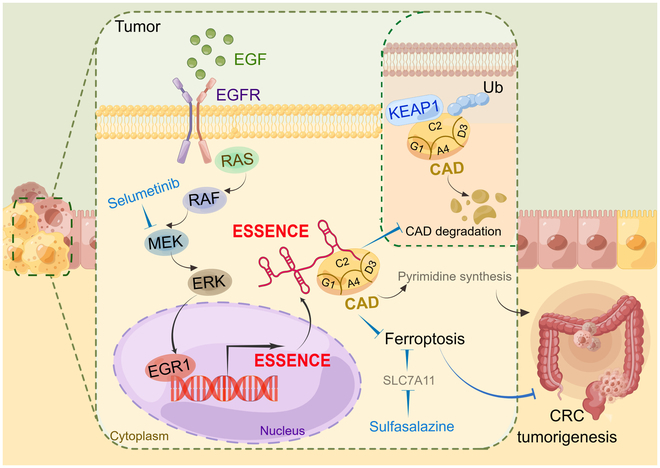
EGF-upregulated lncRNA ESSENCE promotes CRC progression through diminishing KEAP1-dependent CAD degradation. Upon the induction of ESSENCE by activation of the EGFR–rat sarcoma (RAS)/RAF/MEK/ERK–EGR1 axis, CAD is no longer vulnerable to KEAP1-mediated ubiquitination due to ESSENCE’s competitive binding effect. As a result, CAD is stabilized, thereby facilitating CRC tumorigenesis via promoting proliferation and suppressing ferroptosis. The schematic diagram was prepared using Figdraw.

Signal-regulated lncRNAs can exert their biological functions through interactions with proteins, thereby regulating target protein to affect downstream activities to promote cancer progression. Understanding and targeting this regulation could serve as a therapeutic strategy for cancer treatment. Here, we have identified that ESSENCE upregulated by EGFR-MAPK-EGR1 signaling interacts and stabilizes CAD. CAD stabilization could accelerate pyrimidine synthesis and enhance ferroptosis defense, thereby promoting CRC tumorigenesis. Our results reveal that combinatorial targeting of ERK-EGR1 signaling and ferroptosis defense demonstrates promising effects to suppress ESSENCE-high CRC growth (Fig. 8).

### EGF signaling in inducing ESSENCE expression

The EGF/MAPK signaling pathway is notably active in a variety of malignancies, positioning it as a pivotal target for cancer therapeutics. While the activation of MAPK is a common occurrence in numerous cancers, particularly in advanced stages, the consequence of aberrant activation of many MAPK targets is not yet fully understood. We identified a novel molecule—ESSENCE—and characterized its full length regulated by this pathway. When cells are stimulated by the growth factor EGF, EGR1 can be activated through the MAPK signaling pathway [30]. Our studies demonstrate that EGR1 can bind to the promoter region of ESSENCE, providing evidence that EGF signaling is involved in inducing ESSENCE transcriptional expression. EGR1 expression correlates with the progression of several cancers, including prostate cancer and gastric cancer [48,49], and it is involved in proliferation, apoptosis, and matrix degradation. However, its role and downstream targets in CRC remain elusive. We show that both ESSENCE and EGR1 are elevated in CRC and correlated with poor prognosis. Thus, EGF/EGR1 activation can increase the expression of ESSENCE transcription to drive downstream signals involved in CRC progression, adding another role of EGF/EGR1 in regulating tumorigenesis.

### ESSENCE signaling in reducing CAD ubiquitination and degradation

Our studies demonstrate that ESSENCE interacts with CAD and stabilizes CAD through competing with KEAP1, thereby interfering with KEAP1-mediated CAD ubiquitination. CAD is a multifunctional (the trifunctional rate-limiting enzyme of the de novo pyrimidine synthesis pathway) protein catalyzing the first 3 steps of pyrimidine biosynthesis, but its role or regulation in cancer remains not well characterized. Upon urea cycle dysregulation, nitrogen is diverted toward CAD, thereby increasing pyrimidine synthesis [50,51]. CAD plays a crucial role in maintaining cell self-renewal, proliferation, and ferroptosis defense [45,52]. It plays an important role in various cancers and is a lung cancer marker [53]. Also, targeting CAD can inhibit glioblastoma stem cells’ survival, self-renewal, and in vivo tumor initiation [44]. Mutations in EGFR can cause CAD phosphorylation to activate carbon influx through pyrimidine synthesis [34]. Although regulatory mechanisms affecting CAD activity, such as transcription, translation, phosphorylation, oligomerization, and allostery, have been documented [50,54], CAD’s role and regulation in CRC remain not clear. Our data show that EGF signaling (not through EGFR mutation) through ESSENCE expression in CRC can regulate CAD expression via ubiquitination. We describe for the first time that CAD can be regulated by ubiquitination through E3 ligase KEAP1 and that ESSENCE is competing with KEAP1 to bind CAD, preventing CAD–KEAP1 interaction. KEAP1 is a well-known redox-sensitive protein and a unique E3 ligase targeting NRF2, which is involved in maintenance of redox and the progression of many diseases, including diabetes and cancer [55]. KEAP1 instigates NRF2 degradation under unstressed conditions, whereas stimuli that disrupt the redox balance directly alter the thiol groups of KEAP1, thereby inactivating KEAP1 function to cause stabilization of NRF2 [55]. KEAP1 mutations are observed in 11% to 27% of advanced non-small-cell lung cancer, suggesting its role as a tumor suppressor [56]. KEAP1 has only a few characterized substrates. The oncoprotein CAD has the KEAP1-binding sequence as evidenced by our results and is thus added to the new KEAP1 substrate list. Whether KEAP1-mediated CAD degradation can be disrupted by other signals regulated by EGFR/MAPK other than ESSENCE impact remains to be determined. It is possible that other EGF signaling molecules may participate in inactivating KEAP1 since KEAP1 is such a stress sensor [55]. Nevertheless, it makes sense to have a tumor suppressor like E3 ligase KEAP1 to degrade potential oncogenic CAD protein in CRC.

### The ESSENCE–CAD axis is involved in ferroptosis

Previous studies have shown that disrupting DHODH activity leads to extensive mitochondrial lipid peroxidation and triggers ferroptosis [45], suggesting DHODH’s role in ferroptosis defense. Furthermore, a multienzyme complex known as the “pyrimidinosome”, which includes CAD, uridine 5′-monophosphate synthase, voltage-dependent anion-selective channel protein 3, DHODH, and glutamic-oxaloacetic transaminase 1, can enhance DHODH-mediated ferroptosis defense [52]. Given that ESSENCE stabilizes CAD, it is possible that ESSENCE can regulate CAD downstream activities such as ferroptosis. Indeed, we found that ESSENCE knockdown leads to ferroptosis increase and that this process is CAD dependent since CAD expression can revert the ESSENCE knockdown-mediated ferroptosis. ESSENCE knockdown causes ferroptosis with concurrent down-regulation of GPX4 and SLAC7A11, 2 important ferroptosis regulators. It remains to be studied how these 2 regulators are regulated by ESSENCE. Thus, our data identify an ESSENCE–CAD axis involved in ferroptosis defense mechanism and suggest therapeutic strategies by targeting this regulation for CRC treatment. For precision medicine, the choice of ESSENCE–CAD-axis-targeted therapy based on the ESSENCE profile is indeed an appealing therapeutic strategy.

### Combined treatment in ESSENCE-high CRC

EGFR/MAPK signaling plays a vital oncogenic role in CRC, making it an attractive target for therapeutic strategies. For example, MEK is activated in ∼40% of CRC [57]. Selumetinib is an MEK inhibitor that has received Food and Drug Administration approval for the treatment of neurofibroma and CRC [57,58]. We demonstrated that selumetinib has differential treatment efficacies in inhibiting tumor growth depending on the expression levels of ESSENCE in 2 sets of CRC PDX models. The treatment of PDX tumors with high ESSENCE expression with selumetinib can effectively mitigate tumor progression. Conversely, selumetinib has minimal effect on the growth of ESSENCE-low PDX tumors. Our findings may shed light on the reason why not all CRC tumors respond effectively to selumetinib treatment [57,59]. We interpret these outcomes to suggest that the profile of ESSENCE needs to be verified prior to the administration of selumetinib. Given that ERK and ferroptosis are involved in either regulating or relaying ESSENCE activity, respectively, it lends credence to the possibility that a combined therapeutic approach targeting EGFR–ERK activation (selumetinib) along with the induction of ferroptosis (sulfasalazine) could yield superior outcomes in treating ESSENCE-high CRC. As expected, selumetinib plus ferroptosis inducer sulfasalazine as a combination treatment strategy for ESSENCE-high PDX studies demonstrated practicability (Fig. 7D). Our results carry important implications for both the prognosis and the therapeutic implications of CRC with high ESSENCE expression.

Thus, this study uncovers the link between EGF signaling, EGR1 activation, ESSENCE expression, CAD stability, ferroptosis defense, and tumorigenicity. The impacts of EGF/EGR1 in regulating ESSENCE expression, CAD stability via KEAP1, and subsequent CAD-mediated ferroptosis defense to promote tumorigenesis highlight important layers of regulation regarding the expression of ESSENCE during tumorigenicity. Importantly, a rational cancer therapy that targets MEK signaling and induces ferroptosis can be strategically designed for ESSENCE-high cancers.

## Materials and Methods

### Cell culture and transfection

Cell lines were obtained from the American Type Culture Collection and maintained in a humidified incubator containing 5% CO_2_ at 37 °C. HEK293T, HT-29, SW480, SW620, and NCM460 cells were maintained in Dulbecco’s modified Eagle’s medium. HCT116, DLD-1, HCT-8, and HCT-15 cells were cultured in RPMI-1640 medium, while RKO and WiDr cells were maintained in minimum essential medium. The culture medium was supplemented with 10% fetal bovine serum and 1% penicillin/streptomycin. For transient transfection, Polyethylenimine MAX 40,000 (Polysciences Inc., no. 24765-1) or Lipofectamine 2000 (Invitrogen) was employed to transfect small interfering RNAs or the indicated plasmids into the cells. The oligonucleotide sequences are presented in Table S1.

### Patients and tissue samples

CRC tumor samples and matched nontumorous adjacent colorectal tissues were collected from patients who received radical resection at the Cancer Center of Sun Yat-sen University. All patients had a single primary lesion and no neoadjuvant therapy before the surgery. All experimental procedures were granted approval by the Ethics Committee of the Sixth Affiliated Hospital of Sun Yat-sen University.

### Construction of Dox-inducible ESSENCE knockdown cells

Target sequences were selected and used to generate shRNA oligos (as shown in Table S1). The pLKO-Tet-On vector was prepared by codigesting with AgeI and EcoRI enzymes (obtained from New England Biolabs). Following the ligation of vector and shRNA oligos, the resulting DNA was transformed into competent Stbl3 *Escherichia coli* cells, and colonies were screened. After plasmid extraction and virus packaging, CRC cells were then infected with packaged lentivirus, and puromycin was used to select for pools of successfully infected cells. Dox (100 ng/ml) was applied to induce ESSENCE knockdown.

### Mouse models

All mice used in this study were purchased from the Model Animal Research Center of Nanjing University (Nanjing, China). These animals were accommodated in laminar flow cabinets within a specific-pathogen-free environment. The Institutional Animal Care and Use Committee of the Sixth Affiliated Hospital of Sun Yat-sen University granted approval for all animal experiments.

#### Xenograft mouse model

Four-week-old female BALB/c nude mice were employed in the xenograft mouse model. Dox-inducible ESSENCE knockdown HCT116 cells (5 × 10^6^) were harvested and subcutaneously injected into the right flank of the mice. Once tumor volumes reached a range of 30 to 50 mm^3^, all mice harboring tumors were randomly assigned to 2 groups (*n* = 6). The animals received intraperitoneal injections of Dox (at a dosage of 30 mg/kg, Selleck) or a control vehicle every 2 d. Tumor lengths and widths were measured and recorded twice per week. Tumor volume was determined using the formula *V* (mm^3^) = (length × width^2^)/2. At the end of the experiment, all tumors were excised and their weights were recorded.

#### PDX model

The PDX model was conducted as previously described [37]. Briefly, PDX tumors were minced in volume (~1 mm^3^) and subcutaneously implanted into the dorsal flank of NCG (NOD/ShiLtJ-*Prkdc* em26Cd52 *Il2rg* em26Cd22) mice. When the tumor volume reached 30 to 50 mm^3^, mice were assigned randomly into 4 groups: (a) vehicle control, (b) sulfasalazine (100 mg/kg, MedChemExpress), (c) selumetinib (25 mg/kg, MedChemExpress), and (d) sulfasalazine and selumetinib. Sulfasalazine was administered to mice via intraperitoneal injection every 2 d. Selumetinib was orally administrated every 2 d. Mice weights and tumor volumes were measured twice a week until the endpoint. Tumor volume was determined using the formula *V* (mm^3^) = (length × width^2^)/2.

### Quantitative real-time PCR

qRT-PCR was performed following previously described protocol [36]. In brief, total mRNA was isolated using TRIzol Reagent (Invitrogen, no. 15596026) according to the manufacturer’s instructions. Subsequently, 1 μg of RNA was reverse-transcribed into cDNA using ReverTra Ace qPCR RT Master Mix (TOYOBO). qRT-PCR analysis was performed in triplicate in a 20-μl reaction mixture using 2× SYBR Green qPCR Master Mix (BioTool, no. B21203). All gene expression data were normalized to the reference gene β-actin. The primer sequences used for qRT-PCR are presented in Table S2.

### Cell proliferation analysis

The IncuCyte ZOOM Live-Cell Analysis System (Essen BioScience, MI, USA) was employed to record and analyze CRC cell growth. The cells seeded in the plates were placed in the IncuCyte system at 37 °C with 5% CO_2_ for continuous monitoring. Images were captured at ×4 magnification every 2 h, and data analysis was carried out using the Zoom2016 A software (Essen BioScience).

### Cell viability assay

Cell viability was assessed as described previously [60]; 2,000 cells were seeded into 96-well plates and subjected to the specified treatments. Subsequently, the cells were supplemented with 100 μl of medium containing 5% Cell Counting Kit-8 reagent (APExBIO, K1018) for 3 h at 37 °C with 5% CO_2_. Cell viability was determined by assessing the absorbance at 450 nm using a microplate reader.

### Foci formation assay

A total of 300 cells were seeded into the plates and treated as indicated. After being cultured for 10 d, the cells were fixed with 4% formaldehyde for 20 min and stained with 0.5% crystal violet for 30 min. Subsequently, the plates were washed and subjected to quantify the number of foci. Three independent experiments were conducted.

### Luciferase reporter assay

HEK293T cells were seeded into 12-well plates. After adhering to the wall, cells were transfected with a mixture containing 500 ng of firefly luciferase reporter, 10 ng of *Renilla* luciferase reporter, and 500 ng of pcDNA3.1-EGR1 plasmids. In the knockdown treatment, cells were treated with 250 ng/ml Dox for 48 h to induce short hairpin RNA-EGR1 (sh-EGR1) knockdown. After transfection with the plasmids for 48 h, luciferase activity was assessed utilizing the dual-luciferase reporter assay system (Promega, Cat. No. E1910) following the instructions. In brief, the cells were lysed using Passive Lysis Buffer to release the luciferase enzymes. Luciferase Assay Reagent II was added to measure firefly luciferase activity, followed by Stop & Glo Reagent to quench firefly luciferase and measure *Renilla* luciferase activity. The ratio of firefly luciferase activity to *Renilla* luciferase activity was calculated to normalize for transfection efficiency and experimental variability.

### Chromatin immunoprecipitation

The ChIP assay was conducted as previously described [61]. In brief, cells were seeded into 150-mm dishes. Following treatment, cells were harvested and cross-linked at room temperature for 20 min, utilizing 1% formaldehyde, followed by quenching with 0.125 M glycine. The cells were then lysed in ChIP lysis buffer. Following centrifugation, the lysate was treated with nuclear lysis buffer and sonicated with a Diagenode Bioruptor Pico sonicator, employing a 30-s on-and-off cycle for a total of 20 cycles. Following the reservation of 10 μl of the sheared chromatin as input for control samples, the remaining chromatin was immunoprecipitated with EGR1 antibody (CST, Cat No. 4153, 1:100) or control rabbit immunoglobulin G in conjunction with ChIP beads at 4 °C overnight. Immunoprecipitated chromatin was washed sequentially with low-salt buffer, high-salt buffer, LiCl buffer, and Tris–EDTA buffer. The protein–DNA cross-links were digested with proteinase K, and the DNA was purified using a PCR purification kit (Omega). The purified DNA was subjected to qRT-PCR analysis to detect EGR1-binding sites. The primer sets’ details are in Table S2.

### 5′- and 3′-RACE assays

The transcriptional initiation and termination sites of ESSENCE were revealed by performing 5′- and 3′-RACE assays using the SMARTer RACE 5′/3′ Kit (Takara). The 5′- and 3′-RACE-specific primers used for PCR are presented in Table S3.

### RNA–protein pull-down assay

lncRNA ESSENCE and the negative control lncRNA-antisense ESSENCE were transcribed in vitro using TranscriptAid T7 High Yield Transcription Kit (Thermo) as described. The transcribed RNA was labeled with biotinylated cytidine bisphosphate at the 3′ end using Pierce RNA 3′ End Desthiobiotinylation Kit (Thermo). The lncRNA-binding proteins were enriched using the Pierce Magnetic RNA-Protein Pull-Down Kit (Thermo) following the manufacturer’s instructions. The proteins were eluted and analyzed by immunoblot or mass spectrometry analysis.

### Immunoblotting

The immunoblotting was conducted as previously described [8,9]. Total cell lysates were solubilized in lysis buffer containing 50 mM Tris–HCl at pH 7.5, 1 mM EDTA, 150 mM NaCl, 0.1% NP-40, and 0.1% Triton X-100 and supplemented with phosphatase and protease inhibitors. The proteins were separated via sodium dodecyl sulfate–polyacrylamide gel electrophoresis (SDS-PAGE) gels and subsequently transferred onto polyvinylidene fluoride membranes (Millipore). After blocking with 5% nonfat milk for 1 h, the membranes were incubated with the indicated primary antibodies at 4 °C overnight. Afterward, the membranes were washed and incubated with horseradish peroxidase-conjugated anti-rabbit or anti-mouse secondary antibody for 1 h at room temperature. Following multiple washes, an enhanced chemiluminescence system (Bio-Rad) is used to capture chemiluminescence signals from the immunodetection bands on x-ray films.

### Immunoprecipitation

For in vivo exogenous immunoprecipitation (IP), cells were transfected with the indicated plasmids for 48 h. Subsequently, the cells were pre-treated with MG132 (50 μM, MedChemExpress) for 6 h and then harvested. Cell lysis was achieved using IP lysis buffer, consisting of 50 mM Tris–HCl at pH 7.5, 1 mM EDTA, 150 mM NaCl, 0.1% NP-40, and 0.1% Triton X-100 and supplemented with phosphatase and protease inhibitors. Subsequently, the lysates were subjected to an overnight incubation at 4 °C with anti-Flag (DYKDDDDK epitope tag) M2 agarose beads (Sigma-Aldrich, A2220) or anti-Myc magnetic beads (BioTool, B26302). Following incubation, the beads underwent 4 rounds of washing with cold IP lysis buffer before being subjected to immunoblot analysis.

For in vitro IP, TNT Quick Coupled Transcription/Translation systems (Promega, no. L1170) were employed to generate targeted proteins in vitro, following the standard protocols. Specific proteins were pulled down using anti-Myc magnetic beads or anti-Flag M2 agarose beads, followed by immunoblotting with indicated antibodies.

### Protein turnover assay

Cells were seeded into plates and treated as indicated. Subsequently, CHX was introduced into the medium, achieving a final concentration of 160 μg ml^−1^. The cells were then harvested at the designated time intervals following CHX treatment. Finally, the protein levels were assessed through immunoblotting and quantified by ImageJ.

### Ubiquitination assay

For CAD ubiquitination assay, cells were transiently transfected with the indicated plasmids. After 48 h, cells were treated with MG132 (50 μM, MedChemExpress) for 6 h before harvesting. The cells were collected and lysed in denaturing buffer (6 M guanidine-HCl, 0.1 M sodium phosphate buffer/NaH_2_PO_4_, 10 mM imidazole, pH 8.0). Cell lysates were incubated with nickel–nitrilotriacetic acid (Ni-NTA) agarose beads (Invitrogen) at 4 °C overnight. Subsequently, the beads underwent a washing step using Wash Buffer containing 25 mM Tris–HCl and 20 mM imidazole at pH 6.8. Eluted proteins were then analyzed through SDS-PAGE and subjected to immunoblotting using the specified antibodies. Antibodies specific for CAD (Cell Signaling), KEAP1 (Proteintech), Flag-Tag (Sigma), HA-Tag (hemagglutinin tag, Proteintech), Myc-Tag (Cell Signaling), and actin (Sigma-Aldrich) were obtained from the companies as indicated.

### Immunohistochemistry

Immunohistochemistry was carried out as previously described [9,62]. In brief, paraffin-embedded sections were deparaffinized with xylene, followed by hydration in gradient ethanol; 0.01 M sodium citrate was used for antigen retrieval through microwave heating. The sections were then cooled down and incubated with 3% H_2_O_2_ at room temperature for 10 min. After blocking with goat serum for 1 h at room temperature, the sections were incubated with specific primary antibodies at 4 °C overnight. Following this, secondary antibodies were used for incubation for 20 min at room temperature. Immunostaining was visualized using diaminobenzidine, followed by counterstaining with hematoxylin. The following primary antibodies were employed: Ki-67 (Cell Signaling, 9449), PCNA (Abcam, ab29), cleaved caspase-3 (Cell Signaling, 9664), CAD (Cell Signaling, 93925), EGR1 (Cell Signaling, 4153), KEAP1 (Proteintech, 10503-2-AP), SLC7A11 (Proteintech, 26864-1-AP), GPX4 (Proteintech, 67763-1-IG), and p-ERK (Thr202/Tyr204) (Cell Signaling, 4370).

### RNA sequencing, label-free quantitative proteomics, and analysis

Total RNA or protein was extracted from HCT116 cells with or without ESSENCE knockdown. The samples were sent for RNA sequencing or label-free quantitative proteomics performed by Shanghai Majorbio Bio-Pharm Technology Co., Ltd. Sequencing data were subsequently processed to generate expression profiles. The GSEA software provided by the Broad Institute (http://www.broadinstitute.org/gsea/index.jsp) was used to conduct GSEA, following the instructions provided by the Broad Institute.

### Lipid peroxidation assay

Lipid peroxidation assay was performed following a previously described protocol [60]. To quantify lipid peroxidation levels, cells were incubated with 5 μM C11-BODIPY 581/591 (Invitrogen, D3861) for 30 min at 37 °C. Subsequently, the cells were harvested, washed with phosphate-buffered saline, and then assessed using fluorescence-activated cell sorting. The lipid peroxidation levels of the cells were analyzed using the FlowJo_V10 software.

### Cell lysates fractionated by gel filtration

Cell lysates were fractionated based on size-exclusion chromatography, following a previously established method [8,9,36]. In brief, cell lysates were solubilized in lysis buffer containing 50 mM Tris–HCl at pH 7.5, 1 mM EDTA, 150 mM NaCl, 0.1% NP-40, and 0.1% Triton X-100 and supplemented with phosphatase and protease inhibitors. Subsequently, the lysates were passed through a size-exclusion Superose 6 column (GE). Proteins were eluted using phosphate-buffered saline using the GE AKTA avant150 chromatography system, flowing at a rate of 0.4 ml/min. The collected protein fractions were then subjected to SDS-PAGE separation and immunoblotting with the specified antibodies.

### In situ PLA

The standard commercial protocol (Sigma-Aldrich, DUO92101) was followed for conducting PLA [35]. The fixed HCT116 cells were subjected to permeabilization in a 0.5% Triton X-100 solution for 10 min. Subsequently, PLA blocking solution was employed for blocking for 1 h prior to the incubation of the primary antibodies. After overnight incubation in a humidified chamber at 4 °C, the samples were treated with a PLA secondary probe and incubated at 37 °C for 1 h. The ligation process was completed by applying a ligation mix to each sample, incubating it at 37 °C for 30 min. A polymerization mix was used for amplification, and the samples were further incubated at 37 °C for 100 min. After incubation, 1× buffer B was used to wash the samples once for 10 min, followed by another wash with 0.01× buffer B for 1 min at room temperature. Finally, the samples were prepared for imaging by mounting them with Duolink in situ mounting medium containing 4′,6-diamidino-2-phenylindole for 15 min. The proximity ligation signal was captured using a confocal microscope.

### Bioinformatic analysis

Gene expression and overall survival data were sourced from the TCGA database (https://tcga-data.nci.nih.gov/tcga/) and GEO database (https://www.ncbi.nlm.nih.gov/geo/). The protein expression levels of CAD were obtained from the University of Alabama at Birmingham Cancer data analysis portal (https://ualcan.path.uab.edu/). The secondary structure of ESSENCE was analyzed by the RNAfold web server (http://rna.tbi.univie.ac.at//cgi-bin/RNAWebSuite/RNAfold.cgi/). The coding probability of lncRNAs and mRNAs was predicted by CPAT (http://lilab.research.bcm.edu/cpat) and CPC2 (http://cpc2.gao-lab.org/). Transcription factors for ESSENCE were predicted by JASPAR (https://jaspar.genereg.net/).

### Statistical analysis

A Student *t* test or analysis of variance was performed to evaluate the differences between groups, and a paired *t* test was employed for analyzing paired tumor samples of patients, utilizing the SPSS or the GraphPad Prism software. Kaplan–Meier survival analysis was utilized to examine patient survival. Data are presented as mean ± SD from 3 independent experiments, and statistical significance was considered at *P* < 0.05.

## Data Availability

The data reported are available in the article itself or in its online Supplementary Materials.

## Supplementary Materials

Supplementary 1 Figs. S1 to S5 Tables S1 to S3 Table S4 Supplementary resources table
