# Supplementary material for: EGF-Upregulated lncRNA ESSENCE Promotes Colorectal Cancer Growth through Stabilizing CAD and Ferroptosis Defense
Source: Research (Wash D C). 2025 Apr 3;8:0649. doi: 10.34133/research.0649 (PMC11969792; doi:10.34133/research.0649)
Supplement: Supplementary 1 — Figs. S1 to S5 Tables S1 to S3 Table S4 Supplementary resources table [file research.0649.f1.zip › Supplementary Materials-Revised (Clean Version).docx]

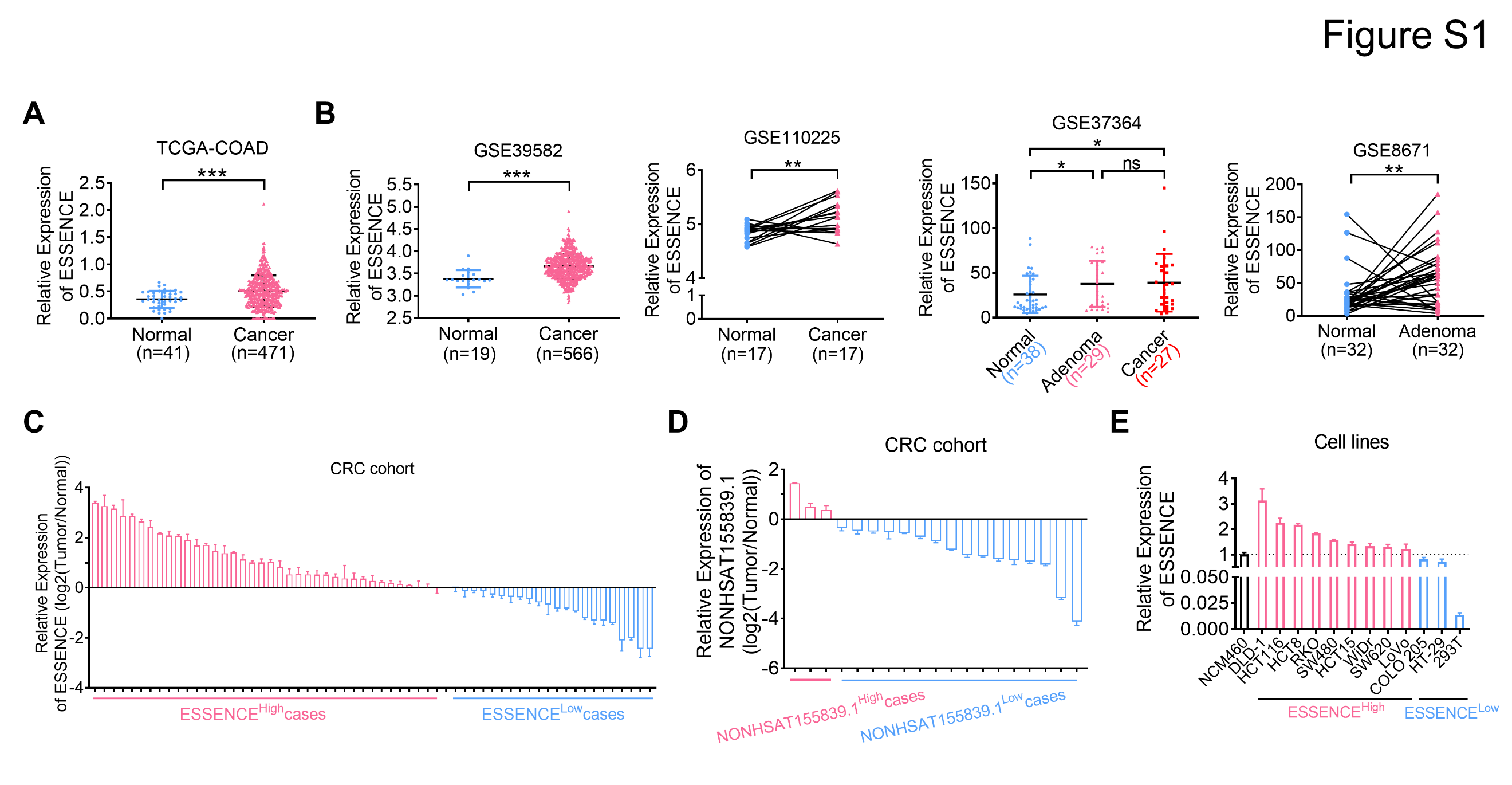
**Figure S1. EGFR/MAPK-upregulated lncRNA ESSENCE is highly expressed in colorectal cancer.**

(A and B) Expression level of ESSENCE in normal mucosa, adenoma and CRC tumor tissues from TCGA (COAD), GSE39582, GSE110225, GSE37364, GSE8671 database.

(C) Relative ESSENCE expression was measured by qRT-PCR in 60 paired samples of colorectal cancer and adjacent non-tumor tissues (CRC cohort). Paired student’s *t* test was conducted.

(D) Relative expression levels of lncRNA NONHSAT155839.1 were measured by qRT-PCR in our CRC cohort consisting of 19 paired samples of colorectal cancer and adjacent non-tumor tissues. Paired student’s *t* test was conducted.

(E) Expression of ESSENCE in different cell lines was detected by qRT-PCR.

Data are presented as means ± SD. **P*<0.05; ***P*<0.01; ****P*<0.001; ns, not significant.


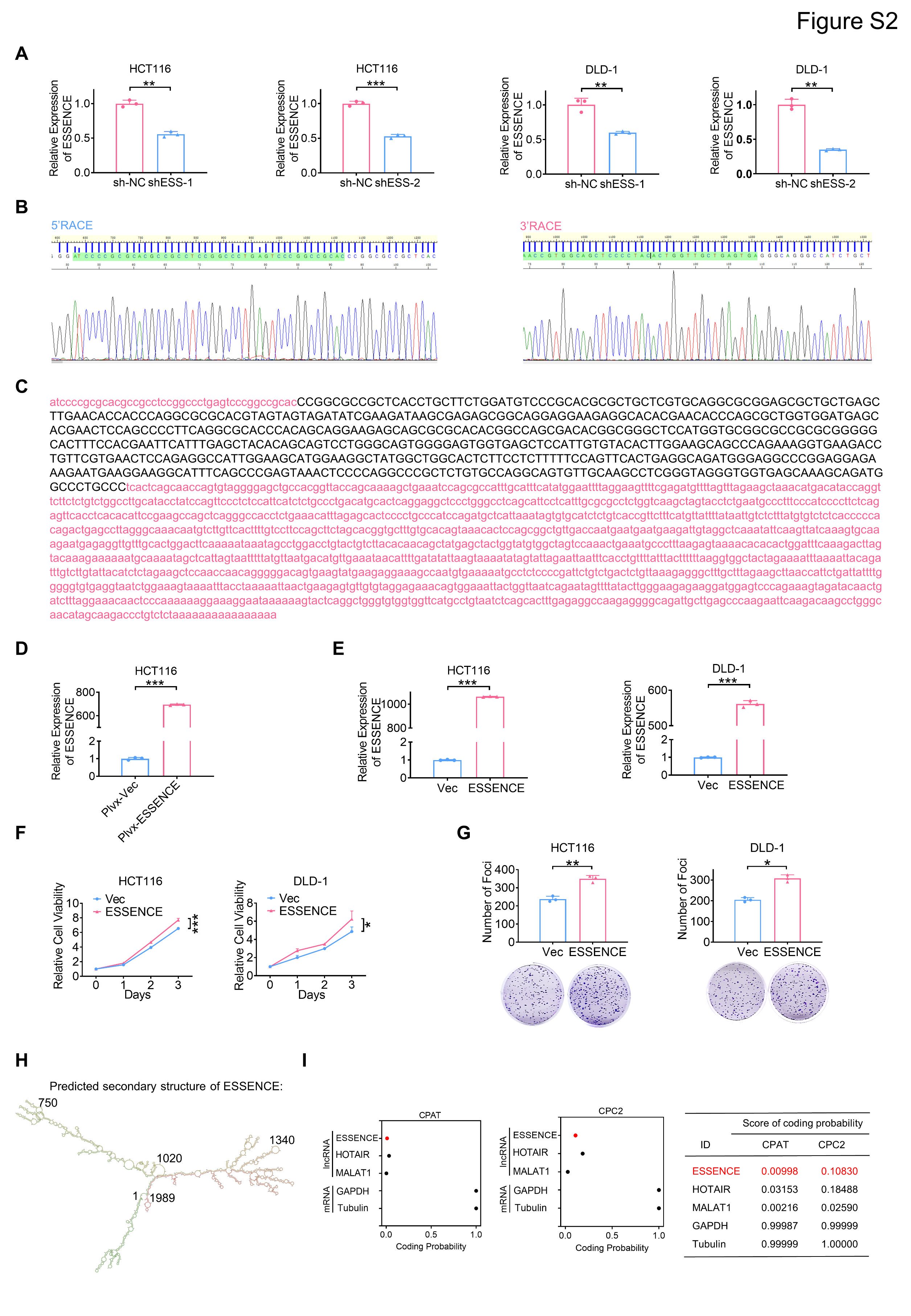


**Figure S2. ESSENCE plays an oncogenic role in regulating CRC cell growth.**

(A) ESSENCE knockdown (Doxycycline-induced) efficiency was measured by qRT-PCR in HCT116 and DLD-1 cells.

(B) Sanger sequencing results of 5'-RACE and 3'-RACE PCR products from the joint part.

(C) The nucleotide sequence of the full-length ESSENCE was shown. The red lowercase letters represent newly identified sequence by RACE assay.

(D and E) Relative ESSENCE expression levels were measured by qRT-PCR in HCT116 and DLD-1 cells after transfected with ESSENCE overexpression plasmids.

(F and G) Proliferation rate and foci formation of HCT116 cells and DLD-1 were assessed after overexpression of ESSENCE.

(H) Predicted secondary structure of ESSENCE using RNAfold webServer.

(I) The predicted coding probability of lncRNAs and mRNAs was analyzed by CPAT and CPC2 websites.

Data are presented as means ± SD. *n* = 3 per group. **P*<0.05; ***P*<0.01; ****P*<0.001; ns, not significant.


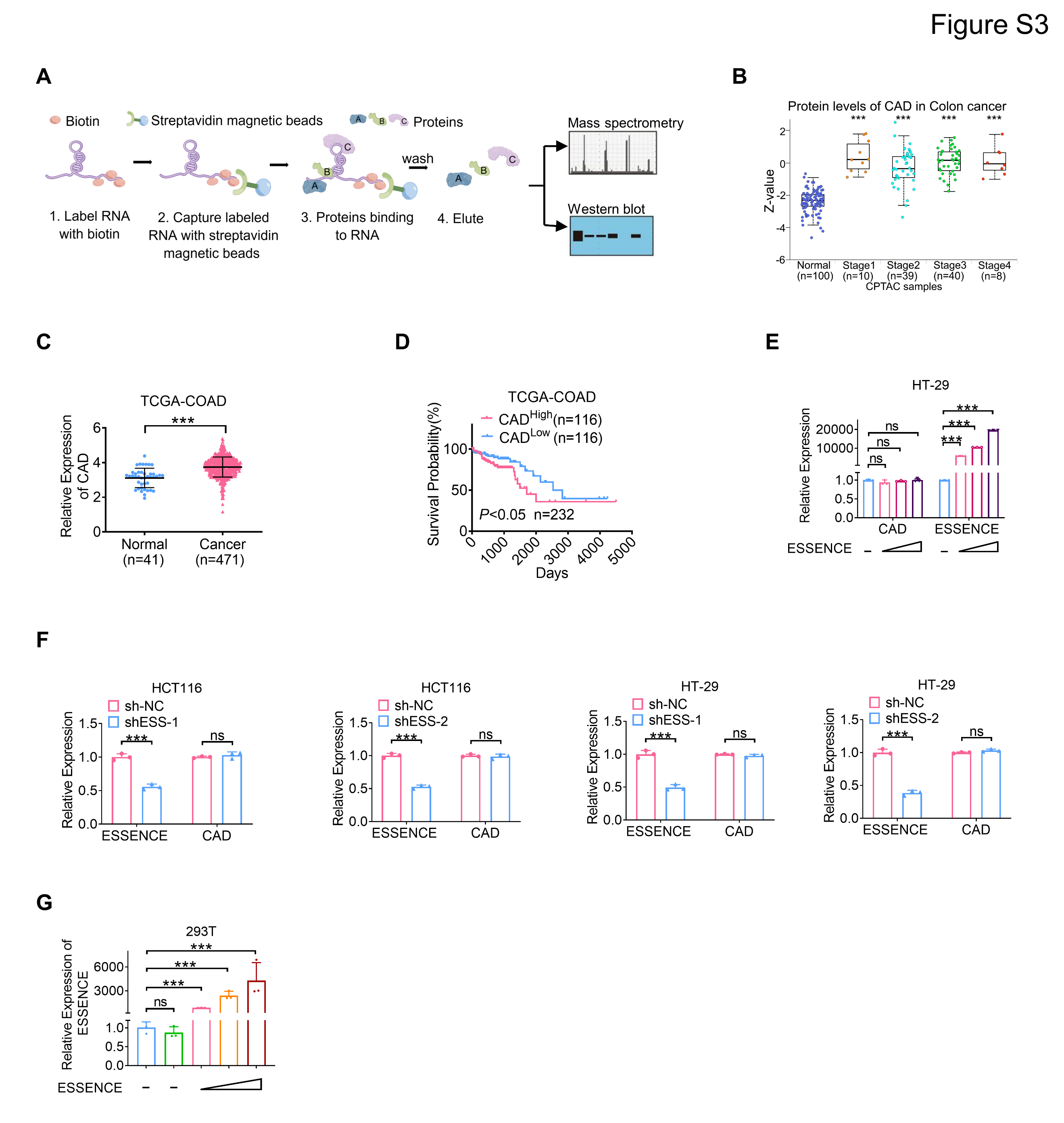
**Figure S3. ESSENCE interacts with CAD and regulates CAD’s steady-state expression.**

(A) Schematic drawing of RNA pull-down assay combined with either mass spectrometry analysis or Western blot detection.

(B) Expression levels of CAD in normal mucosa and different stages of colon cancer from CPTAC database.

(C) Expression levels of CAD in colon cancer and normal tissues from TCGA database (COAD). Unpaired student’s *t* test was conducted.

(D) Kaplan-Meier overall survival (OS) curves were generated based on CAD expression in colon cancer tissues from TCGA database (COAD), with significance testing carried out by log-rank analysis. The quartile expression value (CAD) was used to define the high and low expression group.

(E and F) Relative CAD and ESSENCE expression levels were measured by qRT-PCR in HT-29 and HCT116 cells following ESSENCE overexpression or knockdown.

1. Relative ESSENCE expression levels were measured by qRT-PCR in 293T cells.

Data are presented as means ± SD. *n* = 3 per group. **P*<0.05; ***P*<0.01; ****P*<0.001; ns, not significant.

**
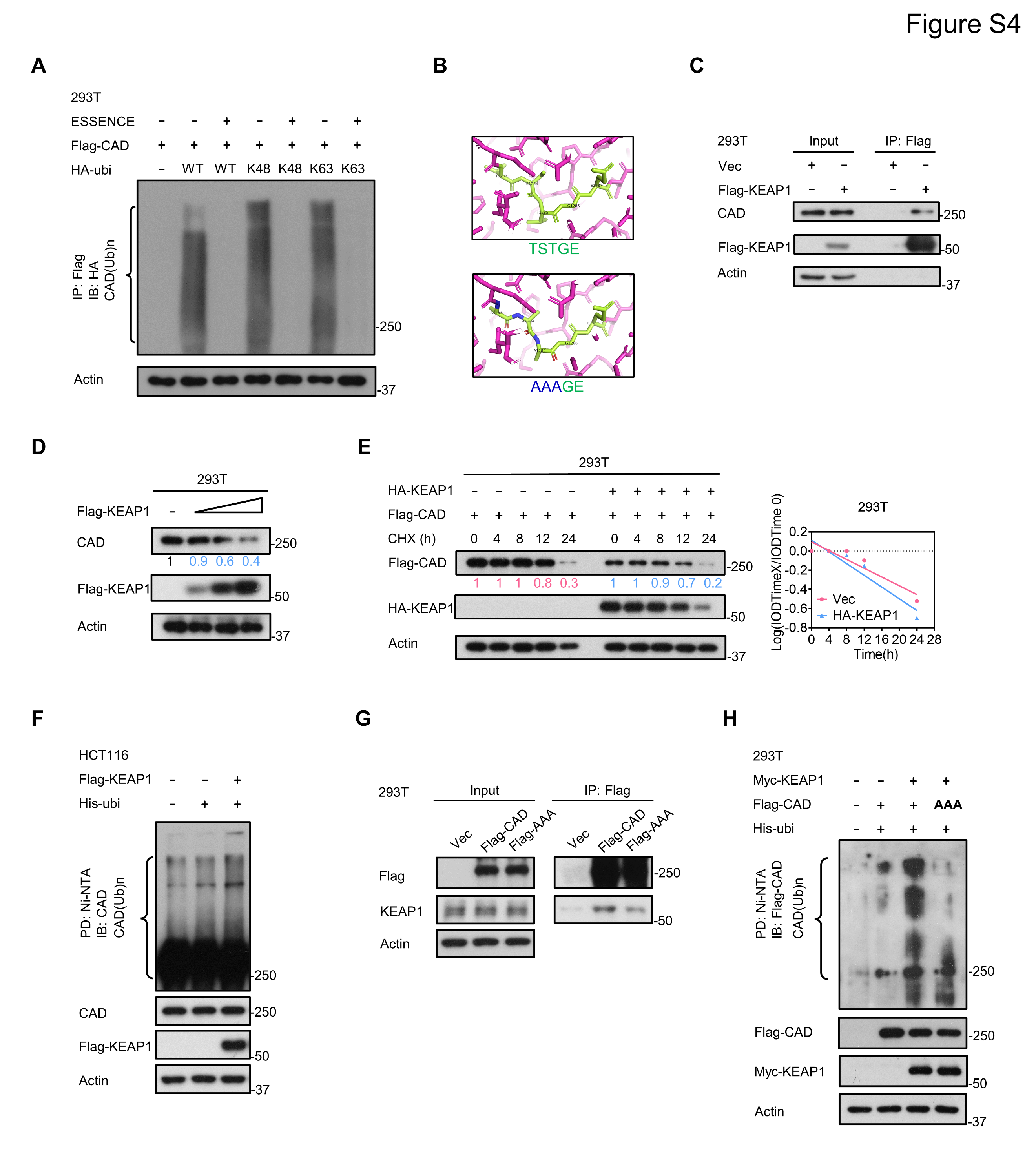
Figure S4. ESSENE inhibits E3 ubiquitin ligase KEAP1-mediated degradation of CAD.**

(A) 293T cells transfected with the indicated plasmids were treated with 50 μM MG132 for 6 hours before harvesting. Cell lysates were immunoprecipitated (IP) with M2 beads and immunoblotted (IB) with indicated antibodies.

(B) Schematic drawing of putative KEAP1-recognized degron (TSTGE) and its mutation (AAAGE) on CAD with Pymol.

(C) Indicated plasmids were transfected into 293T cells, followed by immunoprecipitation with M2 beads and immunoblotting with indicated antibodies.

(D) Representative immunoblot result of CAD steady-state expression following KEAP1 overexpression in 293T cells.

(E) 293T cells were treated with cycloheximide (CHX) (160 µg mL^-1^) for indicated times following KEAP1 overexpression. Lysates were immunoblotted with indicated antibodies. Quantification of CAD turnover rate was analyzed by Image J.

(F) HCT116 cells transfected with the indicated plasmids were treated with 50 μM MG132 for 6 hours before harvesting. Cell lysates were pulled down (PD) with nickel beads (Ni-NTA) and immunoblotted (IB) with indicated antibodies.

(G) Indicated plasmids were transfected into 293T cells, followed by immunoprecipitation with M2 beads and immunoblotting with indicated antibodies.

(H) Cells transfected with the indicated plasmids were treated with 50 μM MG132 for 6 hours before harvesting. Cell lysates were pulled down (PD) with nickel beads (Ni-NTA) and immunoblotted (IB) with indicated antibodies. (AAA: TST to AAA mutant of CAD.)


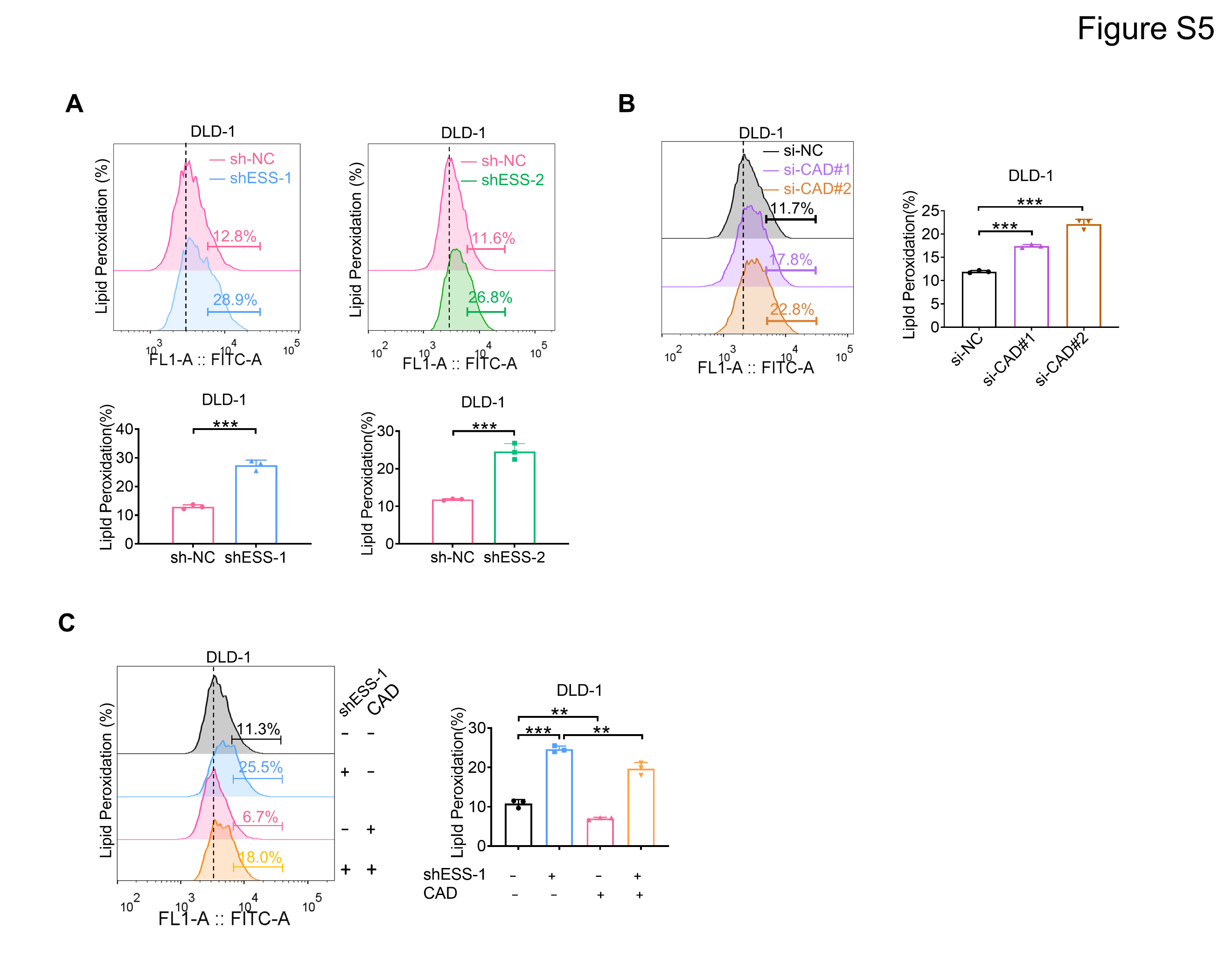
**Figure S5. ESSENCE mediates ferroptosis defence through CAD-DHODH axis.**

(A) Levels of lipid peroxidation were measured following ESSENCE knockdown in DLD-1 cells.

(B) Levels of lipid peroxidation were measured following transfection with CAD siRNAs in DLD-1 cells.

(C) Levels of lipid peroxidation were measured following ESSENCE knockdown and transfection with CAD plasmids in DLD-1 cells.

Data are presented as means ± SD. *n* = 3 per group. **P*<0.05; ***P*<0.01; ****P*<0.001; ns, not significant.

**Supplementary tables**

**Table S1. Oligonucleotide sequences**

| Name | Sequence |
| --- | --- |
| shESS-1 | **TOP:**CCGGGGCATTTCAGCCCGAGTAACTCGAGTTACTCGGGCTGAAATGCCTTTTTG  **Bottom:**AATTCAAAAAGGCATTTCAGCCCGAGTAACTCGAGTTACTCGGGCTGAAATGCC |
| shESS-2 | **TOP:**CCGGGGGTAGGGTGGTGAGCAAACTCGAG TTTGCTCACCACCCTACCCTTTTTG **Bottom:**AATTCAAAAAGGGTAGGGTGGTGAGCAAACTCGAG TTTGCTCACCACCCTACCC |
| sh-EGR1 | **TOP:**CCGGCGGTTACTACCTCTTATCCATCTCGAGATGGATAAGAGGTAGTAACCGTTTTTG  **Bottom:**AATTCAAAAACGGTTACTACCTCTTATCCATCTCGAGATGGATAAGAGGTAGTAACCG |
| si-CAD#1 | CAAGUGCUAGUAGACAAGUTT |
| si-CAD#2 | UGACCAUUGGCAGCUAUAATT |
| siDHODH | GCAUCGAUGGGCUGAUUGUTT |

**Table S2. Primers for qRT-PCR**

| Name | Sequence |
| --- | --- |
| ENST00000415336  (ESSENCE) | F: CCACGAATTCATTTGAGCTACAC  R: CACGAACAGGTCTTCACCTT |
| NONHSAT082934.2 | F: CCGAAGCAGAAATCCATAGAA  R: ACCCTGAGTCCTCACAATAGA |
| NONHSAT155839.1 | F: GGAGCAGGAAAGTGTTTTAG  R: CTGCTGAACAAGAAACTAGGC |
| NONHSAT013049.2 | F: CTTGGCATGGCCTCTTAGAA  R: CCTCCGTGCTGGCAAATA |
| ENST00000439050 | F: GTCTGTCTATTCCTCCCATTGAC  R: GCTGGGCAACATAAACCATAAA |
| MSTRG.60920.1 | F: TCACATTGGTGGGTAGTTACAG  R: CAGTTACAGACAAGGTCCATCA |
| NONHSAT047096.2 | F: ATGACAGGTGAACCCACTTTAG  R: GGTGGAGGAAATGCCTCTTTA |
| ENST00000620266 | F: TCTGTCTATGACCTGAGGTAGG  R: GAAGCCAACAGTCTCCTCTTT |
| ENST00000618776 | F: GAACTCTCGGTCTCTCAGTTTC  R: ATTCTGAAAGGTCTGCAGTGA |
| ENST00000606853 | F: ACTGATGGAGATGTTGGTAGTG  R: GAGACCTGAGTAGACGGTAGT |
| NONHSAT203641.1 | F: CCTGCAAGGTAGAAGAAGTGAG  R: CCCAGCAAGACTGACAGATAAA |
| ENST00000561232 | F: AAGGCACTGTCTGGGTAAAG  R: GTCTATGTCCATCAGCCATCAT |
| NONHSAT072013.2 | F: CTCCTAGCATGGGATGTACTTTAC  R: GGGAATGCACCTTGTTCTACT |
| ENST00000601075 | F: ATTTGGCTCAGCAGTTAACAGA  R: TTGTGTCCTCAGTGGGCTCCT |
| ENST00000555045 | F: CTCACTAACACACAGGGATGG  R: CAGCTAGCAGGAACTGTAGAATAG |
| ENST00000626677 | F: CGCCAACCCTGAGACAAA  R: CATATGACCAGGCAGAGTGAA |
| CAD | F: AAGGAGATTGACGTGGATGC  R: TGGCAGTGATATCTTGTGGG |
| EGR1 | F: GGTCAGTGGCCTAGTGAGC  R: GTGCCGCTGAGTAAATGGGA |
| SP3 | F: GGTTTCACAGGCTCTTCAGATA  R: GGTGTTCCAGAGGCAAGTAA |
| SP1 | F: GCAGGATGGTTCTGGTCAAATA  R: GCTGGAGTAGGTTTGGCATAG |
| β-actin | F: CATGTACGTTGCTATCCAGGC  R: CTCCTTAATGTCACGCACGAT |
| U6 | F: CTCGCTTCGGCAGCACA  R: AACGCTTCACGAATTTGCGT |
| ChIP primers for EGR1 |  |
| S1 | F: TAGCACCAAACGGACTTTTC  R: CAAATGCTGGGAAGCCGA |
| S2 | F: GGCTTCCCAGCATTTGCTCA  R: TGGGTCTAGAGATTGGGAAG |
| S3 | F: ATCTCCCTCCAGGTACCCGC  R: CGTTGGAGAGGGGTTGGAAC |
| S4 | F: ACCCCTCTCCAACGCCAGCT  R: GACGCGAGGTGGCGACGGGA |
| S5 | F: CCGTCGCCACCTCGCGTCC  R: AGAAGCAGGTGAGCGGCGCC |
| Neg | F: CTGGCCAGCAAGGCTGACAG  R: CACTTCGATCCTCTCTTCTAGC |

**Table S3. Primers sequences for Cloning**

| Name | Sequence |
| --- | --- |
| ESSENCE full length | F: ATCCCCGCGCACGCCGCCTC  R: AGAGACAGGGTCTTGCTATGTTGCCCAG |
| ESSENCE promoter | F: ATCTCCCTCCAGGTACCCGC  R: AGAAGCAGGTGAGCGGCGCC |
| EGR1 | F: ATGGCCGCGGCCAAGGCCGAGAT  R: CCAGTAGAGAGGGAGGACTTGGCTCTG |
| SP3 | R: ATGACCGCTCCCGAAAAGCCCG  R: TTACTCCATTGTCTCATTTCCAGAA |
| SP1 | F: ATGAGCGACCAAGATCACTCCATG  R: TCAGAAGCCATTGCCACTGATATT |
| CAD full length | F: ATGGCGGCCCTAGTGTTGGAGGACG  R: CTAGAAACGGCCCAGCACGGTGG |
| CAD AAA mut | F: ATGGCCGCTGCTGGGGAGGTGGCCGGCTT  TGGGGAGAGCCGC  R: CACCTCCCCAGCAGCGGCCATTTCCACAC CCAACACCACGTC |
| CAD-G1 | F: ATGGCGGCCCTAGTGTTGGAGGACG  R: GGGACAGAGGCGCTCAGTCAGC |
| CAD-C2 | F: CCTGGGATTCCCACTCCCGG  R: ACAGTCAACATGCACCTTCAAAGG |
| CAD-D3 | F: ATGACCTCCCAAAAGCTTGTGCG  R: TGAGGGTGGGAGCTGAGGAACAG |
| CAD-A4 | F: GCCCCTGCCACTAGTGAGATGACCA  R: CTAGAAACGGCCCAGCACGGTGG |
| RACE primers |  |
| 5’GSP primer | CACCCTACCCGAGGCTTGCAACACTG |
| 3’GSP primer | GGCTATGGCTGGCACTCTTCCTCTT |
