# Supplementary material for: EGF-Upregulated lncRNA ESSENCE Promotes Colorectal Cancer Growth through Stabilizing CAD and Ferroptosis Defense
Source: Research (Wash D C). 2025 Apr 3;8:0649. doi: 10.34133/research.0649 (PMC11969792; doi:10.34133/research.0649)
Supplement: Supplementary 1 — Figs. S1 to S5 Tables S1 to S3 Table S4 Supplementary resources table [file research.0649.f1.zip › Supplementary resources table.docx]

**Key Resources table**

| **REAGENT or RESOURCE** | **SOURCE** | **IDENTIFIER** |
| --- | --- | --- |
| **Antibodies** | | |
| Rabbit monoclonal anti-CAD | Cell Signaling | Cat# 93925 |
| Rabbit Polyclonal anti-KEAP1 | Proteintech | Cat# 10503-2-AP |
| Rabbit monoclonal anti-EGR1 | Cell Signaling | Cat# 4153 |
| Mouse monoclonal anti-β-Actin | Sigma-aldrich | Cat# A5441 |
| Mouse monoclonal anti-Flag-Tag | Sigma | Cat# F1804 |
| Mouse monoclonal anti-myc-tag | Cell signaling | Cat# 2276S |
| Rabbit Polyclonal anti-HA-Tag | Proteintech | Cat# 51064-2-AP |
| Mouse monoclonal anti-Ki67 | Cell signaling | Cat# 9449 |
| Mouse monoclonal anti-PCNA | Abcam | Cat# ab29 |
| Rabbit monoclonal anti-Cleaved Caspase-3 (Asp175) | Cell Signaling | Cat# 9664 |
| Rabbit Polyclonal anti-SLC7A11 | Proteintech | Cat# 26864-1-AP |
| Mouse monoclonal anti-GPX4 | Proteintech | Cat# 67763-1-IG |
| Rabbit monoclonal anti-Phospho-p44/42 MAPK (Erk1/2) (Thr202/Tyr204) | Cell Signaling | Cat# 4370 |
| **Bacterial and virus strains** | | |
| DH5a Competent cells | Biomed | Cat# BC102 |
| Stbl3 Competent cells | Biomed | Cat# BC108 |
| **Chemicals, peptides, and recombinant proteins** | | |
| Carbenicillin | Biotopped | Cat# C6251 |
| Doxycycline | Selleck | Cat# S4163 |
| Cycloheximide | MDBio, Inc. | Cat# C012-1g |
| MG132 | Selleck | Cat# S2619 |
| EGF | Life Technologies | Cat# PHG0311 |
| Selumetinib | Selleck | Cat# S1008 |
| Sulfasalazine | MedChemExpress | Cat# HY-14655 |
| Polybrene Infection/Transfection reagent | Sigma | Cat# TR-1003G |
| Puromycin 2HCl | Selleck | Cat# S7417 |
| Anti-Flag M2 beads | Sigma-Aldrich | Cat# A2220 |
| Anti-Myc magnetic beads | Selleck | Cat# B26302 |
| Protein A/G PLUS-Agarose | Santa Cruz | Cat# sc-2003 |
| Ni-NTA agarose | Invitrogen | Cat# R90115 |
| Clarity western ECL substrate | Bio-Rad | Cat# 170-5061 |
| TRIzol reagent | Invitrogen | Cat# 15596026 |
| **Critical commercial assays** | | |
| PrimeSTAR Max DNA Polymerase | Takara | Cat# R045B |
| ReverTra Ace qPCR RT Master Mix with gDNA Remover | TOYOBO | Cat# FSQ-301 |
| 2x SYBR Green qPCR Master Mix | Selleck | Cat# B21203 |
| EasyPure HiPure Plasmid MiniPrep Kit | Transgen | Cat# EM111-01 |
| Mut express II Fast Mutagenesis Kit V2 | Vazyme | Cat# C214-02 |
| SMARTer RACE 5'/3' Kit | Takara | Cat# 634858 |
| TranscriptAid T7 High Yield Transcription Kit | Thermo | Cat# K0441 |
| Pierce^TM^ RNA 3’ End Desthiobiotinylation Kit | Thermo | Cat# 20163 |
| Pierce^TM^ Magnetic RNA-Protein Pull-Down Kit | Thermo | Cat# 20164 |
| TNT Quick Coupled Transcription/Translation System | Promega | Cat# L1170 |
| **Deposited data** | | |
| RNA-seq | Gene expression Omnibus (GEO) | GSE37364 |
| RNA-seq | Gene expression Omnibus (GEO) | GSE177606 |
| RNA-seq | Gene expression Omnibus (GEO) | GSE39582 |
| RNA-seq | Gene expression Omnibus (GEO) | GSE110225 |
| RNA-seq | Gene expression Omnibus (GEO) | GSE8671 |
| RNA-seq | Gene expression Omnibus (GEO) | GSE58058 |
| RNA-seq | The cancer Genome Atlas program | TCGA colon cancer and rectal cancer |
| Proteomic | Clinical Proteomic Tumor Analysis Consortium (CPTAC) | CPTAC colon cancer |
| **Experimental models: Cell lines** | | |
| HCT116 | ATCC | Cat# CCL-247 |
| DLD-1 | ATCC | Cat# CCL-221 |
| HT-29 | ATCC | Cat# HTB-38 |
| HCT-8 | ATCC | Cat# CCL-244 |
| RKO | ATCC | Cat# CRL-2577 |
| SW480 | ATCC | Cat# CCL-228 |
| HCT15 | ATCC | Cat# CCL-225 |
| WiDr | ATCC | Cat# CCL-218 |
| SW620 | ATCC | Cat# CCL-227 |
| LoVo | ATCC | Cat# CCL-229 |
| COLO 205 | ATCC | Cat# CCL-222 |
| HEK293T | ATCC | Cat# CRL-1573 |
| **Experimental models: Organisms/strains** | | |
| BALB/c-nu/nu mice | GemPharmatech co., Ltd. | N/A |
| NCG mice | GemPharmatech co., Ltd. | N/A |
| **Oligonucleotides** | | |
| siRNA sequences, see Table S1 | Suzhou GenePharma Co., Ltd. | N/A |
| Primers used for qRT-PCR, see Table S2 | Beijing Genomics Institute | N/A |
| Primers used for cloning, see Table S3 | Beijing Genomics Institute | N/A |
| **Recombinant DNA** | | |
| pCDNA3.1-ESSENCE | Mong -Hong Lee’s Lab | N/A |
| pCDNA3.1-Flag-CAD | Mong -Hong Lee’s Lab | N/A |
| pCDNA3.1-Flag-KEAP1 | Mong -Hong Lee’s Lab | N/A |
| pCDNA3.1-Myc-KEAP1 | Mong -Hong Lee’s Lab | N/A |
| pCDNA3.1-HA-KEAP1 | Mong -Hong Lee’s Lab | N/A |
| pCDNA3.1-Flag-EGR1 | Mong -Hong Lee’s Lab | N/A |
| Tet-pLKO-puro-shESSENCE#1 | Mong -Hong Lee’s Lab | N/A |
| Tet-pLKO-puro-shESSENCE#2 | Mong -Hong Lee’s Lab | N/A |
| **Software and algorithms** | | |
| GraphPad Prism | GraphPad | https://www.graphpad.com/ |
| IBM SPSS Statistics 25.0 | IBM | https://www.ibm.com/cn-zh/spss |
| Gene set enrichment analysis (GSEA) software | GSEA | https://www.gsea-msigdb.org/gsea/index.jsp |
| ImageJ | ImageJ | https://imagej.net/software/imagej/ |
